# Camouflaged Nanozymes with Oxidation‐Promoting Activities Triggering Ferroptosis for Radio‐Immunotherapy

**DOI:** 10.1002/advs.202417370

**Published:** 2025-04-26

**Authors:** Kun Qiao, Yongbiao Huang, Shipeng Ning, Meng Lyu, Jieqiong Xie, Shiyuan Zhang, Xiuxin Lu, Yuan Yu, Wei Jiang, Bo Liu, Kelong Fan, Tong Liu

**Affiliations:** ^1^ Department of Oncological Surgery Harbin Medical University Cancer Hospital Harbin Heilongjiang 150000 China; ^2^ Key Laboratory of Tumor Biotherapy of Heilongjiang Province Harbin Medical University Cancer Hospital Harbin 150000 China; ^3^ Department of Oncology, Tongji Hospital, Tongji Medical College Huazhong University of Science and Technology Wuhan Hubei 430030 China; ^4^ Research Center of Nanomedicine Technology the Second Affiliated Hospital of Guangxi Medical University Nanning 530000 China; ^5^ Academy of Medical Sciences, Tianjian Laboratory of Advanced Biomedical Sciences Zhengzhou University Zhengzhou Henan 450000 China; ^6^ CAS Engineering Laboratory for Nanozyme Key Laboratory of Biomacromolecules (CAS) CAS Center for Excellence in Biomacromolecules Institute of Biophysics Chinese Academy of Sciences Beijing 100101 China; ^7^ Nanozyme Medical Center School of Basic Medical Sciences Zhengzhou University Zhengzhou Henan 450000 China; ^8^ Nanozyme Laboratory in Zhongyuan Henan Academy of Innovations in Medical Science Zhengzhou Henan 450000 China; ^9^ NHC Key Laboratory of Cell Transplantation The First Affiliated Hospital of Harbin Medical University Harbin Heilongjiang 150001 China

**Keywords:** breast cancer, ferroptosis, nanozyme, radioimmunotherapy, radioresistance

## Abstract

Radioresistance presents a substantial obstacle to achieving optimal therapeutic outcomes for breast cancer treatment. In this study, we develop a cancer cell membrane (CM) ‐ coated nanozyme system (MPPC@CM), specifically designed for radioimmunotherapy to address this issue. This innovative system involves the in situ reduction of platinum and palladium on mesoporous silica nanospheres, followed by functionalization with cinnamaldehyde via surface grafting. The CM coating endows the nanozyme with enhanced tumor‐specific targeting capability due to its homing properties. Upon uptake by tumor cells, MPPC@CM catalytically generates O_2_ from H_2_O_2_, mitigating the hypoxic tumor microenvironment and reducing radioresistance.     The intracellular glutathione depletion mediated by Michael addition reactions concurrently disrupts endogenous antioxidant defenses against reactive oxygen species (ROS). This redox imbalance is synergistically amplified through nanozyme‐mediated catalytic activities including both peroxidase‐like and oxidase‐like functions. The resultant massive ROS accumulation establishes a self‐reinforcing oxidative cascade that ultimately induces functional inactivation of glutathione peroxidase 4. The immunosuppressive environment is remodeled by this disturbance in redox balance, which accelerates ferroptosis and increases CD8^+^ T‐cell infiltration and dendritic cell maturation. Overall, this cell membrane‐camouflaged nanozyme holds significant potential to enhance the efficacy of radioimmunotherapy.

## Graphical abstract

This study develops a cancer cell membrane‐coated nanozyme that enhances radiotherapy and reshapes the immunosuppressive microenvironment by generating oxygen in situ and disrupting tumor antioxidant defenses, offering a new strategy for breast cancer radioimmunotherapy.

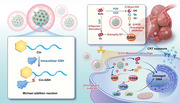

## 1 Introduction

Tremendous advancements in radiotherapy technology have enabled precise dosage delivery to tumor sites with minimal systemic toxicity.^[^ ^1^ ^]^ Radiotherapy is recognized as a standard treatment option for breast cancer.^[^ ^2^ ^]^ As a noninvasive external therapy, postoperative radiation is crucial for eradicating microscopic tumor foci, which helps reduce locoregional recurrence and thus lowers the clinical mortality rate.^[^ ^3^ ^]^ However, specific tumor microenvironment (TME) characteristics contribute to radioresistance, potentially leading to treatment failure.^[^ ^4^ ^]^ The wound healing response, which encompasses vasculogenesis, hypoxia‐inducible factor 1α (HIF‐1α) signaling, and the modulation of cancer‐associated fibroblasts, can contribute to the survival of tumors following radiotherapy. Although radiotherapy can induce an immunogenic cell death (ICD) response, the presence of radioresistant suppressor cell types within the TME frequently leads to immunosuppression.^[^ ^5^ ^]^ This complex interplay within the TME influences the immune response to radiotherapy and fosters radioresistance, leading to tumor recurrence.^[^ ^6^ ^]^ Therefore, strategies need to be developed to prevent tumor recurrence after radiotherapy and achieve effective tumor control.

Tumor cell death from irradiation occurs through two primary mechanisms: direct damage to biomolecules, such as DNA and mitochondria, and indirect damage through the induction of oxidative stress via reactive oxygen species (ROS).^[^ ^7^ ^]^ Oncogenic signaling pathways increase ROS production by activating oxidation‐related enzymes and upregulating the antioxidant glutathione (GSH), which can aid in maintaining redox homeostasis by scavenging ROS.^[^ ^8^ ^]^ However, in the hypoxic conditions of the TME, elevated GSH levels and low oxygen levels can substantially reduce the efficacy of radiotherapy.^[^ ^9^ ^]^ The forms of cell death induced by radiotherapy include apoptosis, necrosis, and autophagic cell death, among which ferroptosis, a nonapoptotic form of cell death that is involved in lipid peroxidation (LPO), is closely associated with the therapeutic efficacy of radiotherapy.^[^ ^9^, ^10^ ^]^ Clinical studies have shown that increasing ferroptosis levels can improve treatment outcomes and prolong progression‐free survival in radiotherapy patients.^[^ ^11^ ^]^ During radiotherapy, externally generated ROS can effectively disrupt the redox balance, resulting in the formation of lipid hydroperoxides (LOOHs) and the subsequent accumulation of LPOs or the regulation of genomic factors that promote ferroptosis. However, GSH can neutralize ROS, thus impeding the reactive metabolism of LPO as a substrate for glutathione peroxidase 4 (GPX4).^[^ ^12^ ^]^ To improve radiosensitization, strategies that can increase ROS levels and deplete GSH to promote LPO‐induced ferroptosis are important. Recent research has indicated that ferrous drugs can induce ferroptosis. However, emerging research on nonferrous compounds has demonstrated the potential to induce ferroptosis through various functions, such as increased ROS production and GSH consumption, thus increasing the efficacy of radiotherapy.^[^ ^13^ ^]^

To address the issues of TME hypoxia and GSH‐mediated radioresistance, enhancing radiotherapy efficacy by applying nanozyme technology for redox status modulation has become an important strategy. Nanozymes composed of noble metals are particularly noteworthy for their exceptional catalytic activities, including peroxidase (POD)‐, oxidase (OXD)‐, catalase (CAT)‐, glutathione peroxidase (GPx)‐, superoxide dismutase (SOD)‐like functions, and metal ion reduction capabilities.^[^ ^14^ ^]^ These nanozymes are distinguished by their excellent electron conductivity, abundant reactive sites, and large surface areas, contributing to their high enzymatic activity.^[^ ^15^ ^]^ Importantly, noble metal‐based nanozymes demonstrate improved catalytic performance due to synergistic effects, making them effective in tumor treatment applications.^[^ ^16^ ^]^ For example, nanozymes containing noble metals such as Pt, Pd, and Mn can alleviate hypoxia in the TME via their CAT‐like activity, thus increasing oxidative stress damage to tumor cells.^[^ ^16^, ^17^ ^]^ Noble metal‐based nanozymes with POD‐like properties can convert H_2_O_2_ into cytotoxic ROS and induce cell death.^[^ ^18^ ^]^ Some nanozymes have shown potential for GSH consumption during tumor treatment.^[^ ^19^ ^]^ Recent advancements in nanozyme design have focused on altering their structure, size, and morphology to increase their catalytic performance.^[^ ^20^ ^]^ In particular, alloying has improved the catalytic capabilities of noble metal‐based nanozymes by increasing the density of active sites for electron transport and optimizing surface atomic structures. Through cascade catalytic processes, bimetallic nanozymes, such as PdMo nanosheets and PtSn nanoclusters, have been created to treat tumors.^[^ ^21^ ^]^ Moreover, by improving energy deposition from ionizing radiation at the tumor site via the photoelectron effect and modulating the TME for tumor suppression, these noble metals in nanozymes can improve treatment outcomes in concert. Therefore, by encouraging ferroptosis through redox balance regulation, the use of noble metal‐based nanozymes for radiosensitization is beneficial.

**Scheme 1:**
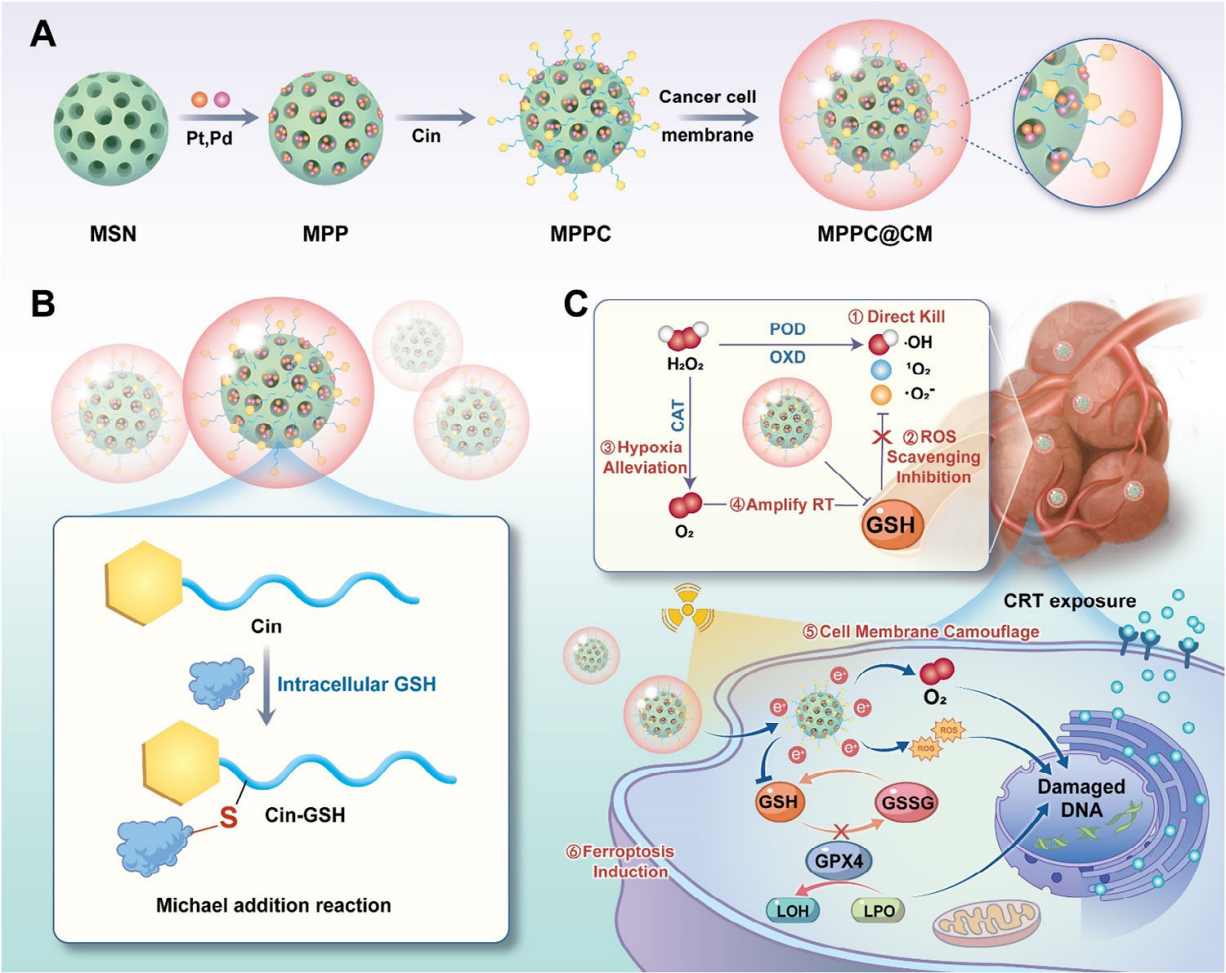
Radiosensitization induced by cancer CM‐camouflaged nanozymes through ferroptosis. A) Synthesis procedure for MPPC@CM. B) Cin targets high levels of GSH through a Michael addition reaction. C) Schematic representation of how MPPC@CM enhances radiotherapy. I) The CM facilitates tumor‐specific targeting and increased cellular uptake. II) MPPC@CM exhibites three enzyme‐like activities, including those of CAT, POD, and OXD, while it inhibites GSH overexpression. III) This leads to an increase in ROS levels and GSH consumption, downregulating GPX4 and triggering ferroptosis. IV) The CAT‐like activity of MPPC@CM mitigates hypoxia in the tumor microenvironment, enhancing ionization damage. V) The inclusion of high‐atomic‐number elements in MPPC@CM enhances energy deposition. These processes enhance DC maturation and facilitate CD8^+^ T‐cell activation, contributing to effective immunotherapy.

Unlike most nanozymes that typically mimic single or dual enzymatic activities, we design a nanozyme termed MPPC@CM with triple enzyme‐like activity, including POD‐/OXD‐/CAT‐like activity, and GSH comsuption ability, which can induce oxidative stress, alleviate hypoxia and deplete GSH, thereby enhancing radiosensitization, leading to ferroptosis (**Scheme** **1**). CAT‐like activity of MPPC@CM reduces hypoxia by converting tumor‐associated H_2_O_2_ into O_2_, lowering hypoxia‐mediated radioresistance and providing a substrate for later OXD‐like reactions. POD‐like action transforms leftover H_2_O_2_ into cytotoxic hydroxyl radicals (·OH), directly causing LPO and ferroptosis. The Michael addition reaction between cinnamaldehyde (cin) and GSH further disables the GPX4 antioxidant system, enhancing LPO accumulation. This cascade forms a self‐reinforcing loop: hypoxia alleviation through CAT‐like activity enhances O_2_‐dependent OXD‐like activity, while GSH depletion (driven by the α,β‐unsaturated ketone structure of cin) and ROS generation (via POD‐like activity) jointly overwhelm redox homeostasis, ultimately leading to increased ferroptosis. This synergy is consistent with recent studies on multi‐enzyme like nanozymes for combinatorial therapy.^[^ ^22^ ^]^

In this study, platinum (Pt)‐palladium (Pd) bimetallic nanozymes are initially synthesized within the pores of mesoporous silica (MSN) via an in situ reduction method. Next, cin is attached to the functional groups of this MSN with PtPd (MPP), forming MPPC. The application of biomimetic camouflaging strategies using cell membranes for nanoparticle encapsulation has significant potential for improving tumor‐targeting efficacy, suggesting a promising paradigm for precision oncology therapeutics.^[^ ^23^ ^]^ Thus, cancer cell membranes (CMs) are used to encapsulate MPPCs to obtain MPPC@CM, leveraging the natural tendency of cancer cells to home to the tumor site. Once localized at the tumor site, the MPPC@CM utilizes the overexpressed GSH for the Michael addition reaction with cin. Moreover, bimetallic nanozymes (BNs) in MPPC@CM demonstrate both POD‐like and OXD‐like activities that convert intracellular H_2_O_2_ into cytotoxic ROS, leading to direct cell death. The hypoxic TME is modified by the CAT‐like property of MPPC@CM, which depletes H_2_O_2_ to produce sufficient O_2_, hence amplifying the therapeutic effects of radiotherapy through a reduction in hypoxia‐inducible factor 1 alpha (HIF‐1α). Thus, MPPC@CM mitigates hypoxia and induces oxidative stress dysfunction by generating ROS and depleting GSH, ultimately triggering ferroptosis by inhibiting GPX4. As a radiosensitizer, MPPC@CM enhances the photoelectron effect due to the presence of high‐atomic‐number elements. These mechanisms also facilitate the exposure of calreticulin (CRT) as an “eat me” signal and the release of high mobility group box 1 (HMGB1) as a “danger signal,” both of which are indicative of ICD. This process facilitates the maturation of dendritic cells (DCs), leading to the activation of CD8^+^ T cells and the conversion of the immunosuppressive “cold tumor” into an immunologically active “hot tumor.” MPPC@CM alleviates hypoxia and enhances beam energy deposition for radiosensitization while also functioning as a GSH scavenger and disruptor of ROS homeostasis to induce ferroptosis. This significantly improves therapeutic outcomes in breast cancer treatment and reduces tumor recurrence.

## 2 Results and Discussion

### 2.1 Synthesis and Characterization of MPPC@CM

**Figure 1:**
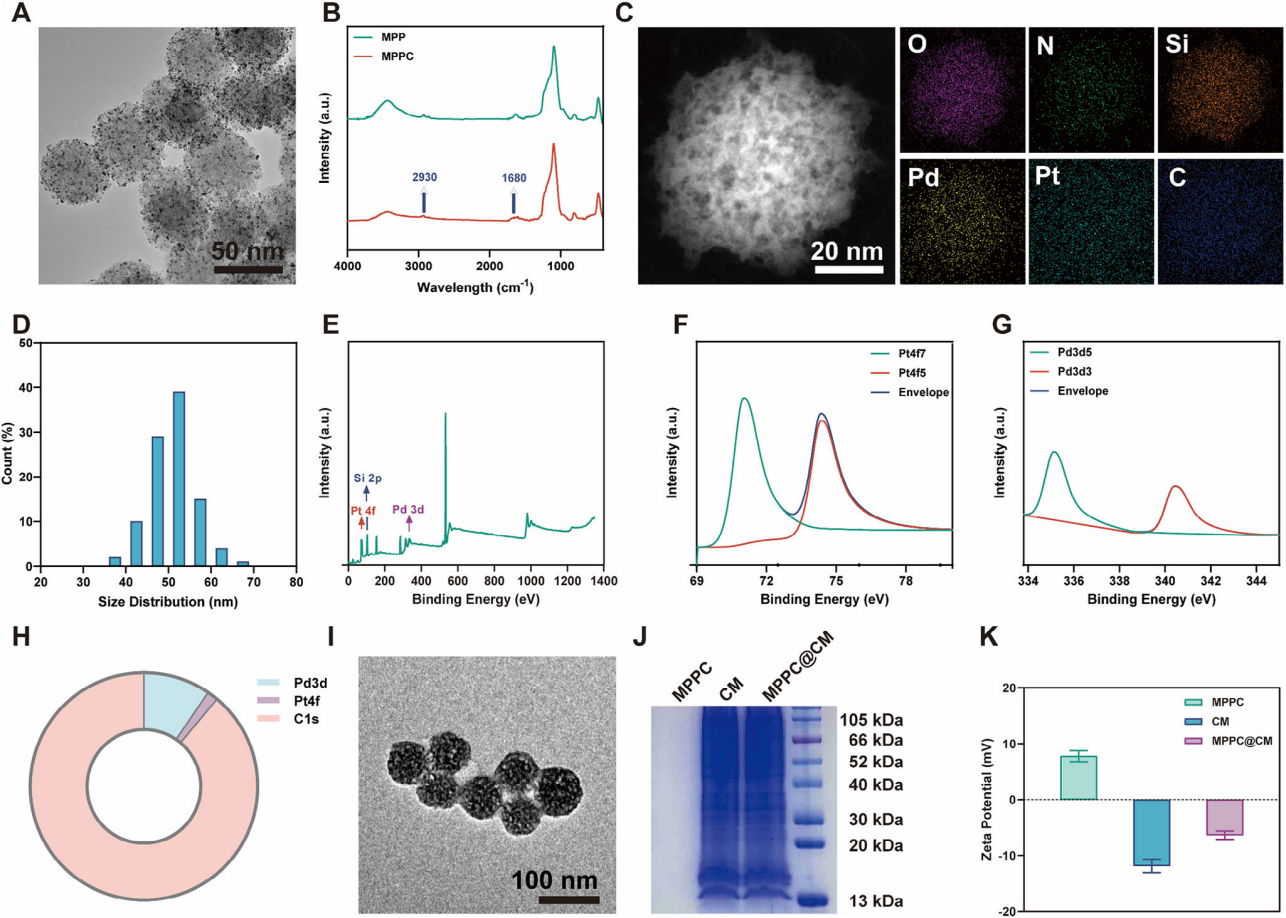
Characterization and physicochemical properties of MPPC@CM BNs. A) TEM images showing the morphology of MPPC BNs. B) FTIR spectra comparing MPP and MPPC, illustrating functional group attachment. C) Elemental mapping of MPPC BNs, displaying the spatial distribution of elements. D) Particle size distribution profiles for MPPC BNs determined via dynamic light scattering. E) Overview of the XPS survey spectrum. High‐resolution XPS spectra for F) Pt and G) Pd, detailing the electronic states and bonding. H) Quantification of atomic percentages in MPPC. I) TEM images depicting the CM‐coated MPPC@CM BNs. J) Comparative analysis of protein expression profiles in MPPC, CM, and MPPC@MC. K) Zeta potential measurements for MPPC, CM, and MPPC@MC, indicating surface charge characteristics.

As outlined in Scheme 1A, the precursors of Pt and Pd were reduced in situ within the pores of the MSNs, followed by the grafting of cin onto the surface groups of the MSNs to form MPPCs. The MPPC was observed via transmission electron microscopy (TEM), which revealed even spherical structures with dark spots on the surface that were thought to be Pt and Pd (**Figure** **1**A). As shown in Figure 1B, Fourier transform infrared (FTIR) spectroscopy revealed distinctive peaks at 1648 and 1288 cm^−1^, which correspond to cin's C‒O and C‒N bonds. The amount of glutaraldehyde grafted onto the MPP was also confirmed via thermogravimetric analysis (TGA). The initial weight loss corresponds to the mass of the loaded nanozyme (23.125%), as shown in Figure S1 (Supporting Information). In comparison, the glutaraldehyde grafted onto the surface (5.553%) was responsible for the subsequent weight reduction. The presence of Pt, Pd, Si, C, and N distributed in these spherical nanoparticles was confirmed by elemental mapping studies, as illustrated in Figure 1C. Dynamic light scattering (DLS) was used to estimate the average particle size at ≈50 nm (Figure 1D). Figure 1E displayed the peaks for Pt 3d and Pd found via X‐ray photoelectron spectroscopy (XPS) analysis.    Figure 1F showed the XPS Pt 4f signals with binding energies at 74.9 and 71.8 eV, corresponding to Pt 4f5/2 and Pt 4f7/2, respectively. The comprehensive Pd 3d high‐definition spectrum in Figure 1G showed two peaks at 334.8 and 340.1 eV, which suggest Pd0 3d5/2 and Pd0 3d3/2, respectively. According to the area comparison, Pd was primarily metallic with a trace amount of divalent elements. The ratio of Pd to Pt in the PtPd deposition was determined to be 34.5% to 65.5%, as shown in Figure 1H. The surface modification of MPPCs involved the application of a CM to exploit the homing effect, as nanoparticles coated with CMs were recognized for their accumulation in tumor tissues. Following the isolation of 4T1 CMs through differential centrifugation, these membranes were coated onto MPPCs via an extrusion method to produce MPPC@CM. Figure 1I showed a thin film outside the nanosphere. Protein expression analysis of MPPC, CM, and MPPC@CM by SDS‒PAGE confirmed that the protein profiles of MPPC@CM were identical to those of CM, as shown in Figure 1J. Figure S2 (Supporting Information) showed that the diameter distributions of MPPC, CM, and MPPC@CM are 51.1, 118.5, and 60.3 nm, respectively. Furthermore, the zeta potential of MPPC@CM shifted from 7.8 to ‐6.4 mV after camouflaging, indicating successful loading (Figure 1K). The average diameter of the MPPC@CM did not significantly change during the 7 days of observation, confirming its stability (Figure S3, Supporting Information).

### 2.2 Enzymatic Activity Evaluation

**Figure 2:**
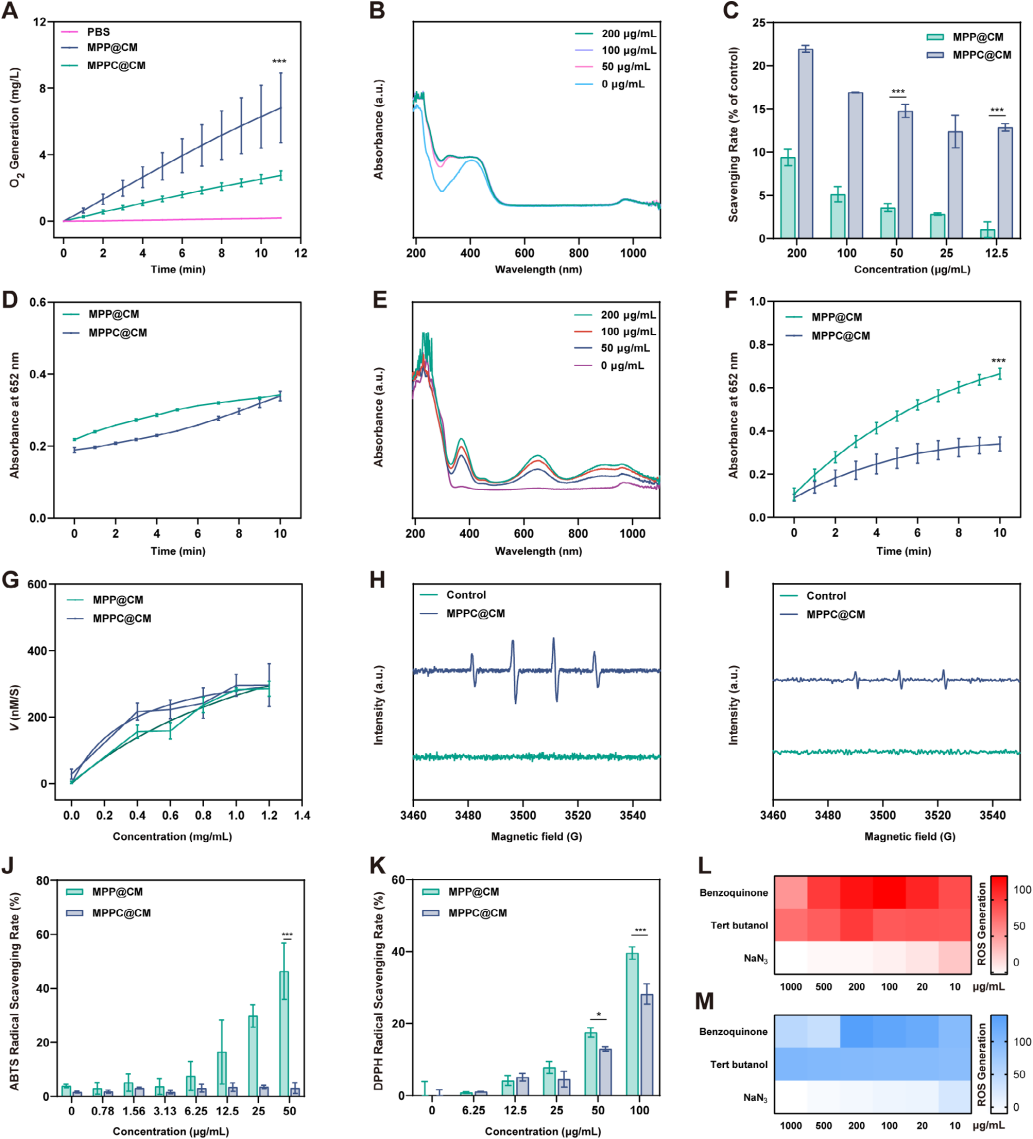
Evaluation of POD‐, OXD‐, and CAT‐like properties of MPPC@CM BNs. A) Oxygen production following the addition of H_2_O_2_ to MPPC@CM. Two‐way analysis of variance (ANOVA) with Tukey's post hoc test, ***p < 0.001. B) Concentration‐dependent UV‒vis absorbance spectra of DTNB solutions treated with MPPC@CM. C) Comparison of the GSH scavenging rates of MPP@CM and MPPC@CM. Two‐way ANOVA with Tukey's post hoc test, ***p < 0.001. D) OXD‐like activity kinetics for MPP@CM and MPPC@CM, as measured via the TMB oxidation assay. E) UV–vis absorbance of various concentrations of TMB in the MPPC@CM group. F) Kinetics of POD‐like activity evaluated via the TMB oxidation assay. Two‐way ANOVA with Tukey's post hoc test, ***p < 0.001. G) Steady‐state kinetic analysis of the POD‐like activity of MPPC@CM with various concentrations of H_2_O_2_. ESR spectra demonstrating the trapping of H) ·OH and I) ·O_2_^‐^ by DMPO. The radical scavenging rate was assessed via J) ABTS and K) DPPH methods. Two‐way ANOVA with Tukey's post hoc test, *p < 0.05, ***p < 0.001. Radical generation activities of L) MPP@CM and M) MPPC@CM were determined using different ROS scavengers. Data was presented as the mean ± SD from three independent experiments.

The hypoxic conditions within the TME often impede the effectiveness of radiotherapy. CAT‐like activity nanozymes that convert H_2_O_2_ into O_2_ can modulate the hypoxic TME and reduce radioresistance. Pt‐based nanozymes have been reported to show CAT‐like activity due to their oxidoreductase properties.^[^ ^24^ ^]^ Therefore, the first step in evaluating the CAT‐like activity of MPPC@CM was to measure O_2_ generation in the presence of H_2_O_2_. Following the addition of MPPC@CM, a time‐dependent increase in O_2_ generation was observed, peaking at 6.83 mg L^−1^ O_2_ at 11 min, as shown in **Figure** **2**A. The catalytic rate constants (*k*_*cat*_) for the CAT‐like activities of MPP@CM and MPPC@CM were calculated to be 0.010438 and 0.004173 s^−1^, respectively (Table S1, Supporting Information). Furthermore, their specific activities were 125.26 U for MPP@CM and 48.10 U for MPPC@CM, highlighting the differences in their catalytic efficiency. GSH is an endogenous antioxidant that maintains the redox balance within tumors, facilitating tumor growth and recurrence. Therefore, decreasing GSH levels is crucial for improving tumor treatment efficacy. The present study explored the GSH scavenging ability of MPPC@CM via a 5,5′‐dithiobis (2‐nitrobenzoic acid) (DTNB) assay. GSH reacts with DTNB to form 2‐nitro‐5‐mercaptobenzoic acid, which displays a characteristic peak at 410 nm. The time‐dependent absorbance spectra of DTNB treated with MPPC@CM revealed that the peak at 410 nm decreased over time, indicating a reduction in GSH levels in response to MPPC@CM. This GSH consumption by MPPC@CM was facilitated through a Michael addition reaction, as highlighted in Figure 2B. Figure 2C presented further comparisons of GSH consumption between MPP@CM and MPPC@CM. The GSH scavenging rate of MPPC@CM exceeded that of MPP@CM across different concentrations, which was attributed to the surface grafting of cin. Moreover, the OXD‐like property of MPPC@CM, which facilitated the production of toxic superoxide anions (·O_2_^−^), was evaluated. Both MPP@CM and MPPC@CM increased the generation of ·O_2_^−^ in a time‐dependent manner, confirming the OXD‐like activity of MPPC@CM, as shown in Figure 2D. Further investigation of the effects of *k*_*cat*_ on OXD‐like activity revealed that the specific activities of MPP@CM and MPPC@CM were 0.0574 and 0.05528 s^−1^, respectively, as shown in Table S2 (Supporting Information).

While H_2_O_2_ is classified as a reactive oxygen species, its cytotoxic effects on tumors are constrained. When a POD‐like enzyme is used, H_2_O_2_ is transformed into the more toxic ·OH, increasing tumor cell death. To assess the POD‐like activity of MPPC@CM, 3,3′,5,5′‐tetramethylbenzidine (TMB), which presents a blue color upon oxidation, was used as a substrate. As depicted in Figure 2E, the UV‒vis spectra revealed an increase in the absorbance at 652 nm upon treatment with MPPC@CM. In comparison, MPP@CM showed greater absorption, suggesting greater POD‐like activity under hypoxic conditions in the presence of H_2_O_2_ (Figure 2F). The kinetics followed typical Michaelis‒Menten behavior, with a Michaelis‒Menten constant (*k_M_*) and maximum reaction rate (*V_max_*) of 0.97 × 10^−3^ M and 0.614 × 10^−7^ M s^−1^, respectively, for MPPC@CM, which was similar to that observed for MPP@CM (Figure 2G). Moreover, the present study explored the effective production of ·OH and ·O_2_^−^ during the reaction by electron paramagnetic resonance (ESR) spectroscopy of MPPC@CM (Figure 2H,I). Since there were some discrepancies between the effects of MPP@CM and MPPC@CM on the GSH consumption ability, which might influence the ROS level, further investigations into the ROS clearance efficiency were conducted using 2,2′‐azinobis (3‐ethylbenzthiazoline‐6‐sulfonic acid) (ABTS, Figure S4, Supporting Information) and 1,1‐diphenyl‐2‐picryl‐hydrazyl (DPPH, Figure S5, Supporting Information). As the concentration increased, radical scavenging rate measured using ABTS decreased in the MPP@CM BNs. The MPPC@CM levels remained low and stable, suggesting that adding cin diminished the ROS clearance capacity of the BNs, increasing their potential to increase oxidative stress (Figure 2J). Colorimetric experiments performed via DPPH showed similar results, which verified the ability of MPPC@CM to scavenge ROS (Figure 2K). Next, we further investigated the ROS species generated. Benzoquinone, tert‐butanol, and NaN_3_ were selected as radical scavengers for singlet oxygen (^1^O_2_), hydroxyl radical (·OH), and superoxide anion (·O_2_^‐^), respectively (Figure S6, Supporting Information). As shown in Figures 2L,M, both MPP@CM and MPPC@CM suppressed the oxTMB absorption peak in the presence of benzoquinone or tert‐butanol, which significantly decreased after the addition of NaN_3_. These results indicated that MPPC@CM eliminated GSH and produced toxic ·OH and ·O_2_‐, which could impair oxidative stress and block ROS clearance. This effect is probably attributable to the ability of cin to scavenge reducible GSH via a Michael addition process.

### 2.3 In Vitro Ferroptosis Promotion and Radiosensitization

**Figure 3:**
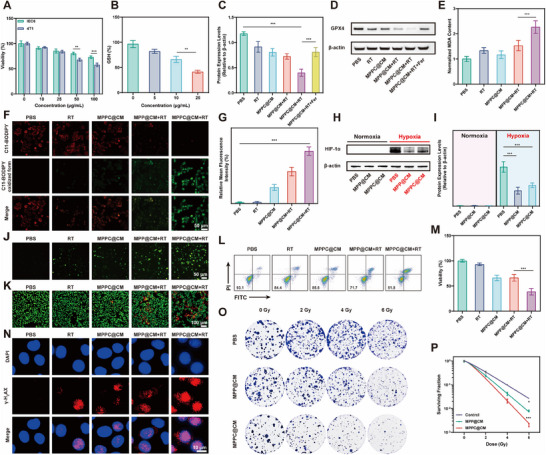
Effects on ferroptosis induction, cell death enhancement, and proliferation inhibition. A) Cytotoxicity in the IEC6 and 4T1 cell lines after coincubation with serial dilutions of MPPC@CM. Two‐way ANOVA with Tukey's post hoc test, **p < 0.01, ***p < 0.001. B) Changes in the intracellular GSH levels in 4T1 cells following treatment with different MPPC@CM concentrations. One‐way ANOVA with Tukey's post hoc test, **p < 0.01. C) Relative GPX4 expression levels in the treatment groups and D) western blot analysis. One‐way ANOVA with Tukey's post hoc test, ***p < 0.001. E) MDA concentrations in various treatment scenarios. One‐way ANOVA with Tukey's post hoc test, ***p < 0.001. F) CLSM images and G) mean fluorescence intensity (MFI) analysis of LPO levels in 4T1 cells evaluated via C11‐BODIPY. One‐way ANOVA with Tukey's post hoc test, ***p < 0.001. H) Western blot results and I) associated relative expression levels of HIF‐1α in cells. One‐way ANOVA with Tukey's post hoc test, ***p < 0.001. J) CLSM images showing ROS production and K) live/dead staining. L) Flow cytometry data showing the rates of cell apoptosis in various treatment groups. M) Cell viability under different experimental conditions. One‐way ANOVA with Tukey's post hoc test, ***p < 0.001. N) Fluorescence imaging of γ‐H_2_AX foci formation. O) Optical images from a colony formation assay and P) corresponding survival curves generated via the “multitarget single‐hit” approach. Two‐way ANOVA with Tukey's post hoc test, ***p < 0.001. Results were presented as the mean ± SD from three independent experiment.

Owing to their remarkable enzyme‐like characteristics, these nanozymes can be used to induce ferroptosis and enhance radiosensitization in vitro. First, a hemolysis assay was conducted to evaluate the biosafety of MPPC@CM. As shown in Figure S7 (Supporting Information), no significant hemolysis occurred at a concentration of 100 µg mL^−1^ MPPC@CM. We then assessed the cytotoxic effects of MPP@CM and MPPC@CM on IEC6 and 4T1 cells, respectively. As shown in **Figure** **3**A, MPPC@CM was more cytotoxic toward 4T1 cells over IEC6 cells, likely due to its capacity to camouflage the CM.

On the other hand, at the same concentrations, MPP@CM had a reduced cell‐killing efficiency, as depicted in Figure S8 (Supporting Information), which was attributed to its excessive ability to scavenge ROS. Further analysis focused on the impact of the cell membrane coating on cellular uptake. FITC‐labeled MPPCs and MPPC@CM were coincubated with 4T1 cells and visualized via confocal laser scanning microscopy (CLSM). Increased cellular uptake was suggested by the data in Figure S9 (Supporting Information), which revealed increased green fluorescence intensity in the MPPC@CM group. A GSH detection kit was then used to track the GSH levels. A concentration‐dependent decrease in the GSH level was shown in Figure 3B, which might be related to the Michael addition process that cin in MPPC@CM facilitates.

The essential function of GSH in preserving redox balance inside the TME means that its depletion can result in the inactivation of GPX4, leading to the accumulation of LPO and thus triggering ferroptosis. A western blot analysis was performed to evaluate this, and the results demonstrated that RT alone resulted in the overexpression of GPX4, as shown in Figure 3C,D. Interestingly, GPX4 expression was significantly reduced under MPPC@CM+RT treatment. However, with the addition of ferrostatin‐1 (Fer‐1), GPX4 levels were restored above those observed in the MPPC@CM+RT group, indicating that the downregulation of GPX4 was related to ferroptosis. TEM images revealed that the number of mitochondria in 4T1 cells treated with MPPC@CM+RT was considerably reduced, with cristae collapsing, which was the same morphology as ferroptosis (Figure S10, Supporting Information). Malondialdehyde (MDA), the end product of LPO, was quantified via a thiobarbituric acid colorimetric assay. As indicated in Figure 3E, MDA levels were elevated in the MPPC@CM+RT group. Furthermore, C11‐BODIPY, a fluorescent probe sensitive to LPO, was employed to detect LPO accumulation. The results in Figure 3F,G showed that green fluorescence representing the oxidized form of C11‐bodipy increased in the MPPC@CM+RT group, confirming that LPO was stimulated under these conditions.

Hypoxia significantly decreases the efficacy of radiotherapy; however, the findings indicated that MPPC@CM, which has CAT‐like activity, may offer a viable solution to this issue. This study examined the upregulation of HIF‐1α, which is linked to adverse effects postirradiation and facilitates cell survival, across various treatment groups. As depicted in Figures 3H,I, HIF‐1α protein expression was low under normoxic conditions but increased under hypoxic conditions. Treatment with both MPP@CM and MPPC@CM was found to reduce HIF‐1α protein levels, indicating alleviation of hypoxia. CLSM images of HIF‐1α in 4T1 cells further confirmed that MPPC@CM effectively alleviated hypoxia (Figure S11, Supporting Information). Next, ROS production was verified via a DCFH‐DA probe, which emits green fluorescence in the presence of ROS. The MPPC@CM group presented green fluorescence, as displayed in Figure 3J, which was explained by its POD‐like characteristics. Surprisingly, the fluorescence intensity of the MPPC@CM+RT group was greater than that of the other groups, suggesting a considerable increase in ROS production.

Inspired by the exceptional enzyme‐like features of MPPC@CM shown in vitro, we conducted live/dead staining and flow cytometry analysis to evaluate cell apoptosis. As shown in Figure 3K,L, the RT alone group exhibited reduced cell apoptosis, but the MPPC@CM+RT group presented a significantly increased apoptosis rate, with only 51.8% of the cells remaining viable. This indicated the highest cell‐killing rate among all the tested groups. Cell viability tests via a CCK8 kit corroborated these findings, revealing that only 41% of the cells remained viable following MPPC@CM+RT treatment, as shown in Figure 3M. Since radiotherapy directly leads to DNA damage, γ‐H_2_AX staining was performed in all the treatment groups. As shown in Figure 3N, weak red fluorescence appeared in the RT alone group, whereas increased red fluorescence intensity was observed in the MPP@CM+RT group. The MPPC@CM+RT group presented the greatest degree of DNA damage to 4T1 cells.

Furthermore, a clonogenic formation assay was performed to evaluate the effects of the treatments on cell survival, with the results fitted via a “multitarget single‐hit” model. As depicted in Figure 3O,P, RT alone moderately reduced 4T1 cell survival, but adding MPP@CM significantly increased the inhibition rate. The number of colonies decreased dramatically with MPPC@CM treatment in a dose‐dependent manner, and the sensitizer enhancement ratios were calculated to be 1.11 and 1.37. These results demonstrated that both MPP@CM and MPPC@CM induced ferroptosis to improve radiosensitization in vitro. Furthermore, MPPC@CM showed an enhanced radiosensitization effect, attributed to its capacity to eliminate GSH and inhibit the removal of oxygen free radicals, a process augmented by the grafting of cin. This elevation in oxidative stress substantially contributes to its radiosensitization efficacy.

### 2.4 Immunostimulation Response In Vitro

**Figure 4:**
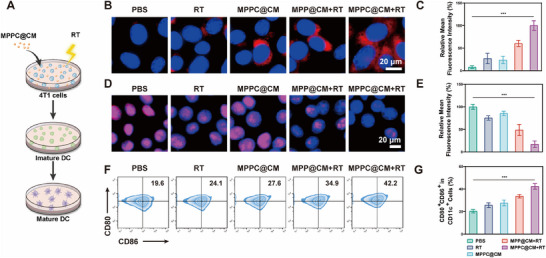
In vitro, MPPC@CM combined with radiotherapy was used to induce ICD. A) Schematic illustration of the DC maturation experiment. CLSM images displaying B) CRT exposure and C) corresponding MFI quantification. One‐way ANOVA with Tukey's post hoc test, ***p < 0.001. D) CLSM images showing HMGB1 release from the nucleus and E) corresponding MFI quantification. One‐way ANOVA with Tukey's post hoc test, ***p < 0.001. F) Flow cytometry analysis of DC maturation and G) the percentage of mature DCs under various treatment conditions. One‐way ANOVA with Tukey's post hoc test, ***p < 0.001. Results were presented as the mean ± SD from three independent experiment.

The effectiveness of radiotherapy is often limited by suboptimal ICD efficiency. The current investigation examined CRT exposure and HMGB1 secretion in each treatment group to determine whether MPPC@CM improved ICD induction in vitro (**Figure** **4**A). The MPPC@CM group presented significantly greater levels of CRT exposure on the cell membrane, as evidenced by the CLSM images in Figure 4B and the associated mean fluorescence intensity (MFI) data in Figure 4C. Moreover, a faint red fluorescence signal of HMGB1 in the cell nucleus was evident in the MPPC@CM+RT group, suggesting the release of HMGB1 from the cell nucleus into the extracellular matrix (Figure 4D). MFI quantification further verified these results (Figure 4E). In subsequent experiments, the capacity of MPPC@CM+RT to encourage DC maturation was assessed. Supernatants from 4T1 cells following different pretreatments were coincubated with bone marrow‐derived DCs from BALB/c mice. The flow cytometry plots in Figure 4F,G demonstrated that MPPC@CM alone increased the fraction of mature DCs from 19.6% to 24.1%, indicating a mild impact on immune activation. The combination of MPPC@CM with radiation significantly enhanced DC maturation, reaching 42.2%, which was 2.16 times greater than that of the control group. These results confirmed that MPPC@CM+RT can significantly enhance ICD induction and promote DC maturation.

### 2.5 Tumor Targeting and Hypoxia Modulation Assessment

**Figure 5:**
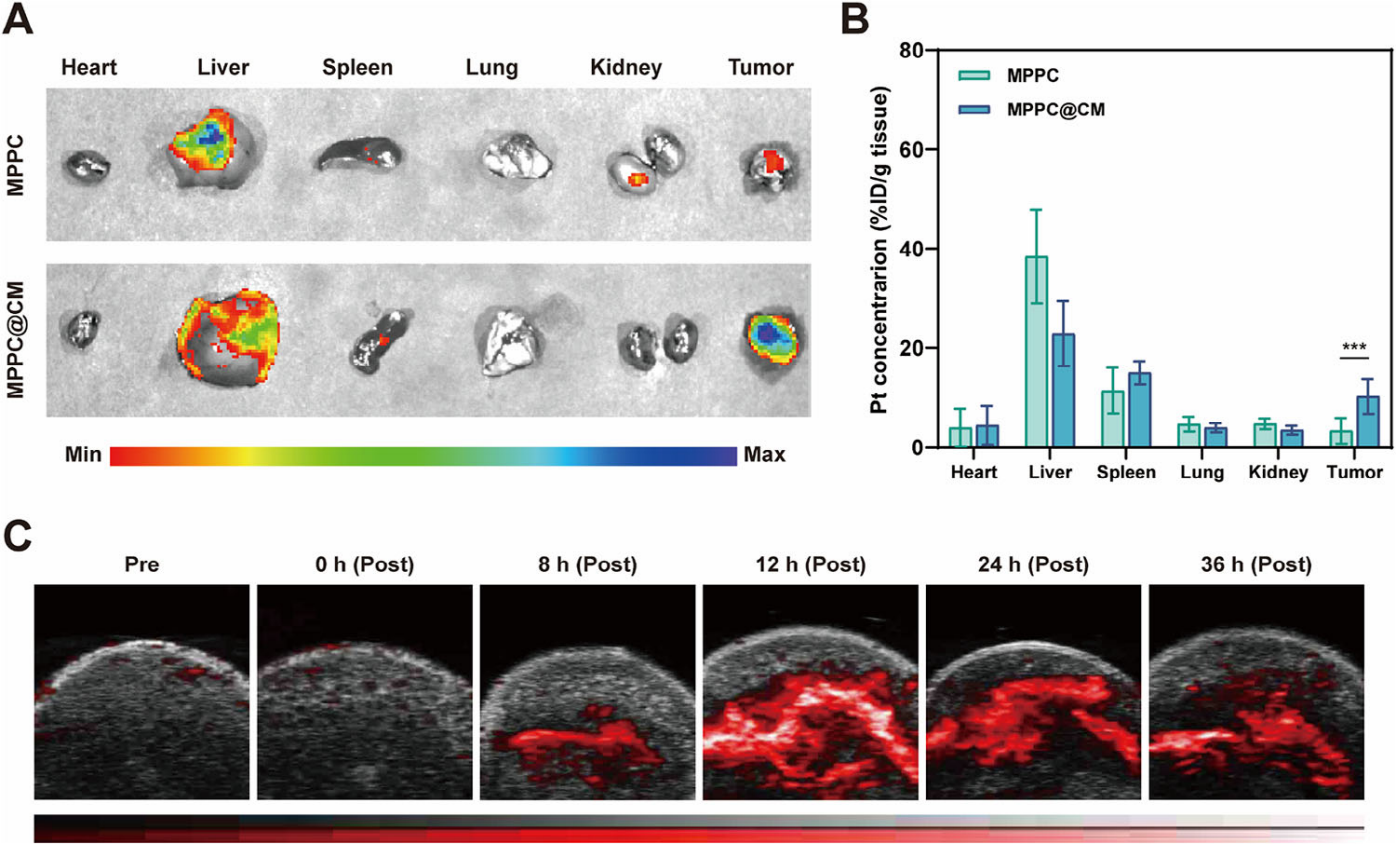
Homing effect and hypoxia modulation of MPPC@CM. A) Ex vivo fluorescence imaging of key organs and tumors in mice 24 h after receiving intravenous injections of either MPPC or MPPC@CM. B) Quantitative biodistribution analysis of the main organs and tumors 24 h postinjection. Two‐way ANOVA with Tukey's post hoc test, ***p < 0.001. C) PA imaging at various time points. Results were shown as mean ± SD from 5 mice of each group.

CM coating is a novel tumor‐targeting strategy that takes advantage of the homotypic binding properties of cancer cells. The distribution of fluorescence intensity from Cy5‐labeled MPPCs and MPPC@CM was analyzed in 4T1 tumor‐bearing mice to confirm the tumor‐targeting capability of MPPC@CM coated with the 4T1 CM (**Figure** **5**A). After treatment, major organs and tumors were harvested from each group for *ex vivo* fluorescence imaging. Surprisingly, the MPPC group presented mild signals in the tumors but a significant fluorescence concentration in the liver and spleen. However, compared with those in the MPPC group, the tumors in the MPPC@CM group presented significantly greater fluorescence intensity, suggesting that the CM coating improved tumor accumulation. The in vivo distribution of the nanozyme was further analyzed, confirming an increased concentration of MPPC@CM in tumor tissues compared with MPPC alone (Figure 5B). Since MPPC@CM can alleviate hypoxia in vitro through its CAT‐like activity, photoacoustic (PA) imaging was utilized to monitor real‐time intratumoral oxygen levels. As shown in Figure 5C and Figure S12 (Supporting Information), the sO_2_ level increased 12 h after intravenous injection of MPPC@CM, with strong signals persisting for up to 24 h. However, the O_2_ concentration decreased 48 h after injection because of elimination by the immune system. Immunofluorescence staining of HIF‐1α, shown in Figure S13 (Supporting Information), revealed a more pronounced reduction in green fluorescence intensity, indicating the presence of more HIF‐1α‐positive regions 24 h after MPPC@CM injection than after MPPC injection, highlighting the increased tumor accumulation caused by the CM coating.

### 2.6 Antitumor Efficacy of MPPC@CM‐Enhanced Radiotherapy Via the Induction of Ferroptosis

**Figure 6:**
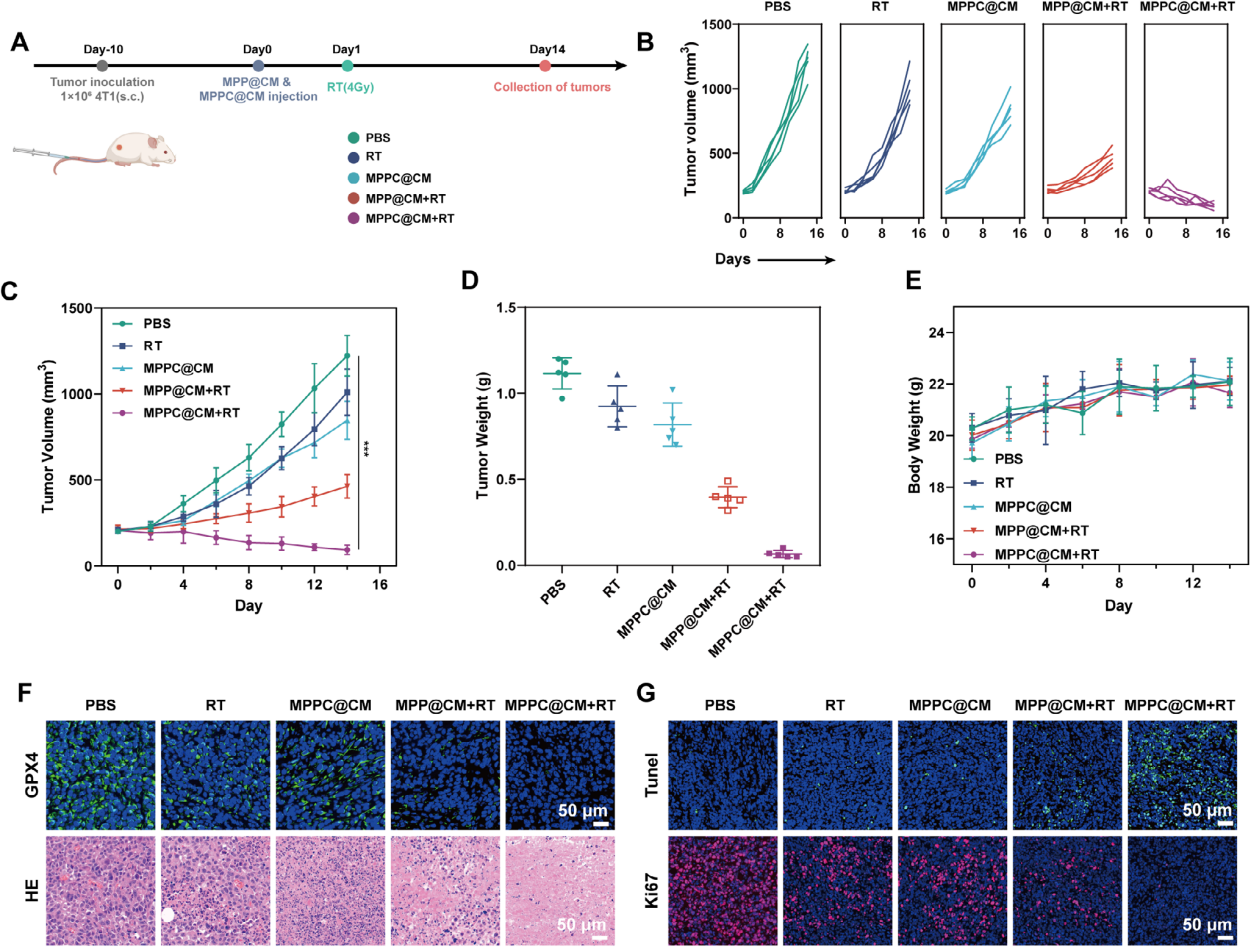
Antitumor efficacy evaluation. A) Schematic illustration of the treatment procedure used in the MPPC@CM+RT group. B) Individual tumor growth trajectories, C) collective tumor growth curves, D) tumor weights, and E) body weight changes under various treatment conditions. Two‐way ANOVA with Tukey's post hoc test, ***p < 0.001. F) Immunofluorescence staining for GPX4 and HE staining. G) TUNEL staining and Ki67 immunofluorescence staining images across different treatment groups. Results were shown as mean ± SD from 5 mice of each group.

Motivated by the ability of MPPC@CM+RT to kill cancer cells in vitro, this study evaluated its anticancer efficacy in vivo. Initially, 14 days after the administration of PBS, MPP@CM, or MPPC@CM via the tail vein, the toxicity of these compounds in healthy mice was assessed via biochemical tests and organ injury monitoring. Blood routine and biochemical parameters related to liver and kidney functions were not significantly different among the groups. Furthermore, major organs stained with hematoxylin and eosin (H&E) presented no evident damage or abnormalities, indicating that MPP@CM and MPPC@CM did not cause severe systemic damage (Figure S14, Supporting Information). This study then established a subcutaneous 4T1 tumor model in BALB/c mice to assess antitumor effectiveness. As indicated in **Figure** **6**A, the mice in the MPPC@CM+RT group were intravenously injected with MPPC@CM and subsequently subjected to X‐ray irradiation the following day. During the experiment, the tumor volume in the PBS control group increased, reaching 1223 mm^3^ by day 14 (Figure 6B,C). Radiotherapy alone had a marginal inhibitory effect on tumor proliferation.

The MPP@CM radiosensitization technique showed substantial antitumor efficacy. The tumor weight in each group corresponded with the data presented in Figure 6D. No significant changes in body weight were observed among the groups over the study period (Figure 6E). Immunofluorescence analysis of dissected 4T1 tumors revealed a substantial reduction in GPX4 expression in the MPPC@CM+RT group, indicating significant ferroptosis (Figure 6F), which was further confirmed by immunohistochemistry analysis (Figure S15, Supporting Information). Furthermore, the results of western blot analysis verified the downregulation of GPX4 after treatment with MPPC@CM+RT (Figure S16, Supporting Information). While TUNEL and Ki67 staining, as shown in Figure 6G, demonstrated that MPPC@CM+RT significantly triggered apoptosis and inhibited proliferation, H&E staining confirmed nuclear pyknosis and tissue necrosis in tumors treated with MPPC@CM+RT. Thus, MPPC@CM+RT therapy substantially reduced tumor development, demonstrating its potential for successful tumor eradication.

### 2.7 Abscopal Effects of MPPC@MC‐Mediated Radiosensitization and PD‐L1 Checkpoint Blockade

**Figure 7:**
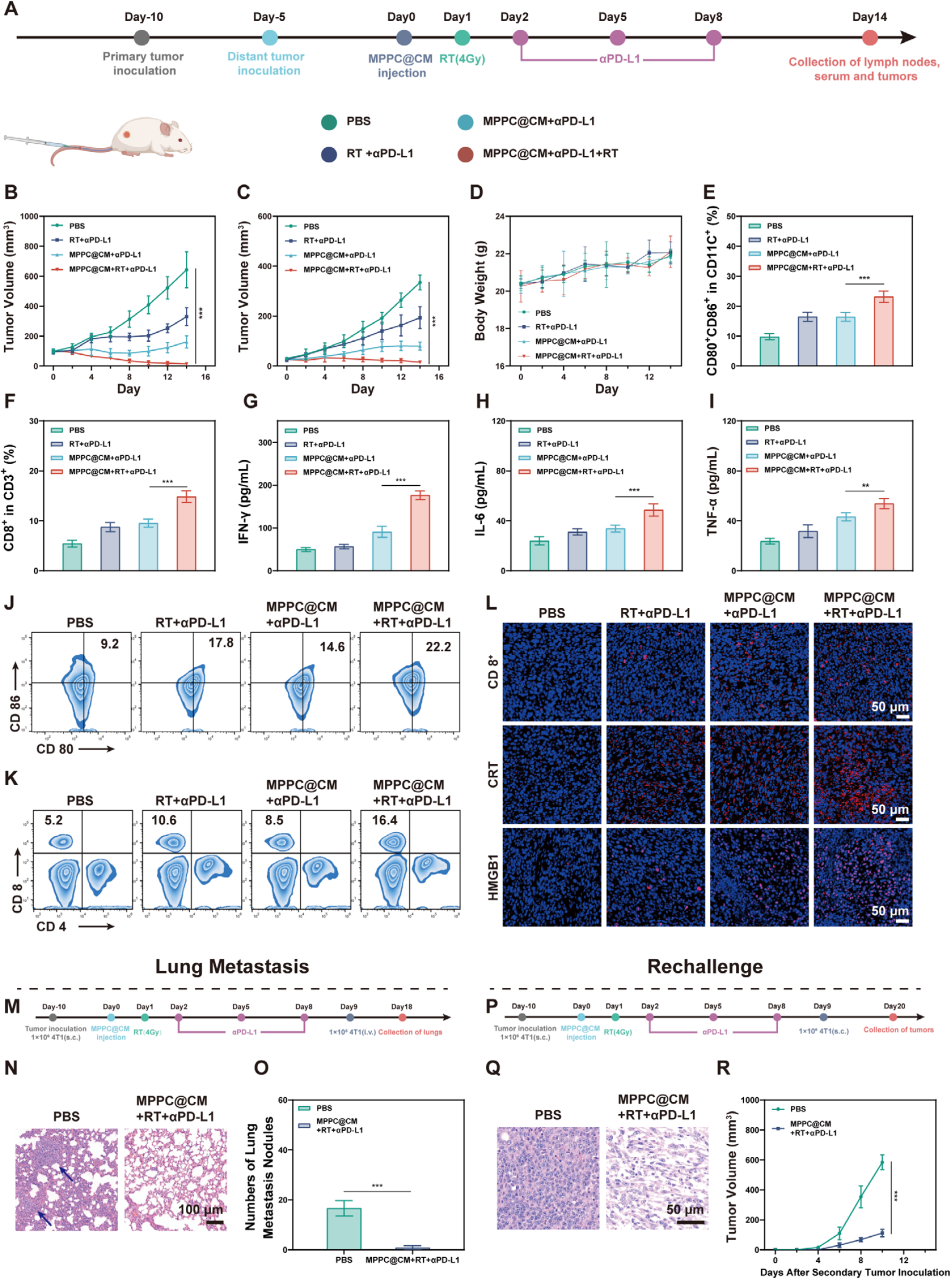
Treatment efficacy of MPPC@CM‐mediated radiosensitization combined with PD‐L1 antibody therapy in a bilateral tumor model. A) Schedule of bilateral tumor model establishment and treatment procedures. Growth curves for B) primary tumors and C) distant tumors. Two‐way ANOVA with Tukey's post hoc test, ***p < 0.001. D) Variations in body weight over time. E) Quantification of CD80^+^CD86^+^ expression on CD11c^+^ cells in the lymph nodes. One‐way ANOVA with Tukey's post hoc test, ***p < 0.001. F) Levels of CD8^+^ CD3^+^ T cells in tumor tissues. One‐way ANOVA with Tukey's post hoc test, ***p < 0.001. G) Measurements of IFN‐γ, H) IL‐6, and (I) TNF‐α cytokine levels across different groups. One‐way ANOVA with Tukey's post hoc test, ***p < 0.001. J) Flow cytometry plots illustrating DC maturation and K) CD8^+^ T‐cell populations in 4T1 tumors. L) Fluorescence microscopy images showing staining for CD8^+^ cells, CRT, and HMGB1. M) Schematic of the therapeutic schedule for a lung metastasis model. N) HE staining of lung tissues and quantification of O) the number of lung metastasis nodules. Two‐way ANOVA with Tukey's post hoc test, ***p < 0.001. P) Schematic of the therapeutic schedule for a tumor rechallenge model. Q) H&E staining of rechallenged tumors. R) Tumor volume in the two groups. Two‐way ANOVA with Tukey's post hoc test, ***p < 0.001. Results were shown as mean ± SD from 5 mice of each group.

Since metastasis remains among the leading causes of cancer‐related death, there has been an increasing interest in the use of PD‐1/PD‐L1 checkpoint inhibitors in conjunction with other treatments, including radiation. This study used a bilateral 4T1 tumor model to assess the effectiveness of PD‐L1 inhibition when combined with MPPC@CM‐enhanced radiation. Initially, a primary tumor was inoculated in the left hip, followed five days later by secondary tumor implantation in the right hip. In accordance with the treatment protocol outlined in **Figure** **7**A, primary and secondary tumor volumes were monitored, as depicted in Figure 7B,C. There was no apparent difference in body weight between the groups (Figure 7D), and these results showed that MPPC@CM‐augmented RT plus anti‐PD‐L1 therapy not only eliminated the primary tumor but also significantly suppressed the development of the secondary tumor, whereas RT plus anti‐PD‐L1 therapy only moderately controlled tumor growth. The in vivo ICD effect was assessed through immunofluorescence staining for CRT and HMGB1, which revealed a significant increase in these markers in the MPPC@CM+RT+aPD‐L1 group compared with those in the PBS group (Figure 7L). This study further examined DC maturation, as DCs are essential for antigen presentation to T cells and their subsequent activation. The flow cytometry data indicated that RT in conjunction with anti‐PD‐L1 facilitated DC maturation (Figure 7E,J), with the proportion of mature DCs in the lymph nodes reaching a maximum of 22.2% in the MPPC@CM‐treated group, which enhanced RT and anti‐PD‐L1. The infiltration level of CD8^+^ T cells in tumors was also assessed (Figure 7F,K), with CD8^+^ T‐cell percentages reaching 16.4% under MPPC@CM+RT+aPD‐L1 treatment, which was 1.55 times greater than that observed in the RT+anti‐PD‐L1 group. Immunofluorescence staining of tumor CD8^+^ T cells yielded similar findings (Figure 7L). The levels of various immune‐related cytokines in the serum, including IFN‐γ, IL‐6, and TNF‐α, were measured via ELISA and were found to increase following MPPC@CM+RT+aPD‐L1 treatment (Figure 7G–I). These data indicated that MPPC@CM+RT+aPD‐L1 treatment caused a significant immune response, effectively suppressing tumor growth.

The substantially improved anticancer efficacy led this investigation to examine the effects of this method on tumor metastasis inhibition and rechallenge. Following the experimental protocol displayed in Figure 7M, the lungs were excised for hematoxylin and eosin (H&E) staining and nodule assessment. As shown in Figure 7N and Figure S17 (Supporting Information), HE staining of the lungs revealed severe metastasis in the PBS group, whereas no evident pulmonary nodules were found in the MPPC@CM+RT+aPD‐L1 group. Compared with the lungs in the control group, which demonstrated multifocal pulmonary consolidation with unclear structure, the surface of the lung tissue in the treatment group was covered by a smooth serosa with no apparent abnormalities. However, reports of minor lymphocytic infiltration surrounding blood vessels were rare. Following treatment, substantially fewer metastatic nodules were observed (Figure 7O). A tumor model with rechallenge was then established (Figure 7P). Tumors were collected after the monitoring period, and tumor volume was measured following secondary tumor implantation. As shown in Figure 7Q,R, H&E staining revealed a disorganized cellular arrangement and a significant decrease in tumor cell density in tissues treated with MPPC@CM+RT+aPD‐L1, indicating that this treatment effectively inhibited secondary tumor growth and induced tumor cell necrosis. MPPC@CM+RT+aPD‐L1 treatment might promote innate and adaptive immune responses, establishing an efficient immunological memory response for tumor suppression, recurrence prevention, and metastasis inhibition.

## 3 Conclusion

The present study developed a multi‐enzymatic tumor‐targeting nanozyme, MPPC@CM, intended for radiosensitization through the activation of ferroptosis. This nanosystem consisted of Pt and Pd nanozymes, which were deposited in situ on the MSN, with cin grafted onto the surface and finally coated with a cell membrane. It possesses CAT‐, POD‐, and OXD‐like properties and can consume GSH via Michael addition reaction. With a 4T1 CM coating, these MPPC@CM BNs leveraged the homing properties of the 4T1 cells to accumulate specifically at tumor sites. The CAT‐like activity of MPPC@CM decomposes tumor‐associated H_2_O_2_ into O_2_, alleviating hypoxia within the TME through an oxygen supply and suppressing radioresistance.

Furthermore, the elevated atomic number of the integrated metals improved radiation deposition. GSH, which is essential for preserving redox equilibrium in the tumor microenvironment, was consumed by the Michael addition of cin to the nanozyme surface. This disruption of redox homeostasis by the generation of abundant toxic ROS through POD‐ and OXD‐like activities and the inhibition of ROS clearance, led to deactivation of the GPX4 enzyme. This cascade intensifies LPO and ultimately triggers ferroptosis in tumor cells. The application of MPPC@CM synergistically improved the efficacy of radiotherapy by disrupting redox homeostasis. Interestingly, when combined with anti‐PD‐L1 therapy, MPPC@CM enhanced radiotherapy inhibited tumor growth and abrogated tumor metastasis. Furthermore, the practical use of MPPC@CM in improving radiotherapy highlights its viability, as it aligns well with current, in‐depth therapeutic strategies and offers a promising avenue for advancing cancer treatment modalities. This synergistic effect of enhanced ferroptosis and immunotherapy suggests that MPPC@CM has substantial potential for clinical translation, potentially setting the stage for the next generation of radiotherapy treatments.

## Conflict of Interest

The authors declare no conflict of interest.

## Supporting information



Supporting Information

## Data Availability

The data that support the findings of this study are available from the corresponding author upon reasonable request.
