# Supplementary material for: Camouflaged Nanozymes with Oxidation‐Promoting Activities Triggering Ferroptosis for Radio‐Immunotherapy
Source: Adv Sci (Weinh). 2025 Apr 26;12(22):2417370. doi: 10.1002/advs.202417370 (PMC12165144; doi:10.1002/advs.202417370)
Supplement: Supplementary file 1 — Supporting Information [file ADVS-12-2417370-s001.docx]

**Supporting Information**

**Camouflaged Nanozymes with Oxidation-Promoting Activities Triggering Ferroptosis for Radioimmunotherapy**

*Kun Qiao, Yongbiao Huang, Shipeng Ning, Meng Lyu, Jieqiong Xie, Shiyuan Zhang, Xiuxin Lu, Yuan Yu, Wei Jiang, Bo Liu*, Kelong Fan*, Tong Liu**

K. Qiao., S. Zhang., X. Lu., Y. Yu. and T. Liu.

Department of Oncological Surgery, Harbin Medical University Cancer Hospital, Harbin, Heilongjiang Province, 150000, China. Email: liutong@hrbmu.edu.cn

K. Qiao.

Key Laboratory of Tumor Biotherapy of Heilongjiang Province, Harbin Medical University Cancer Hospital, Harbin, 150000, China.

T. Liu.

NHC Key Laboratory of Cell Transplantation, The First Affiliated Hospital of Harbin Medical University, Harbin, Heilongjiang Province, 150001, China.

Y. Huang., M. Lyu., and B. Liu.

Department of Oncology, Tongji Hospital, Tongji Medical College, Huazhong University of Science and Technology, Wuhan 430030, Hubei, China. Email: boliu888@tjh.tjmu.edu.cn

S. Ning., and J. X.

Research Center of Nanomedicine Technology, the Second Affiliated Hospital of Guangxi Medical University, Nanning 530000, China.

K. Fan

CAS Engineering Laboratory for Nanozyme, Key Laboratory of Biomacromolecules (CAS), CAS Center for Excellence in Biomacromolecules, Institute of Biophysics, Chinese Academy of Sciences, Beijing 100101, China.

Nanozyme Medical Center, School of Basic Medical Sciences, Zhengzhou University, Zhengzhou 450000, Henan, China.

Nanozyme Laboratory in Zhongyuan, Henan Academy of Innovations in Medical Science, Zhengzhou 450000, Henan, China. Email: fankelong@ibp.ac.cn

W. Jiang.

Academy of Medical Sciences, Tianjian Laboratory of Advanced Biomedical Sciences, Zhengzhou University, Zhengzhou 450000, Henan, China.

**Experimental Section**

**Materials:** Methanol, chloroplatinic acid, palladium chloride, 3-aminopropyltriethoxysilane, cinnamaldehyde, tetraethyl orthosilicate (TEOS), ammonium chloride (NH_4_Cl), 3-aminopropyltriethoxysilane, cetyltrimethylammonium bromide (CTAB), chloroplatinic acid, cinnamaldehyde, and palladium chloride were purchased from Sigma‒Aldrich.

**Synthesis of MSNs, MPPs, MPPCs, MPP@CM and MPPC@CM**

To synthesize mesoporous silica nanoparticles (MSNs), 1 g of CTAB was dissolved in 50 mL of deionized water. The pH of this mixture was subsequently adjusted to 10 by adding NH_4_Cl solution dropwise under continuous stirring at 50 °C, after which 1 mL of TEOS was added. Next, the solution was stirred in a N_2_ atmosphere at 40 °C for 4 hours. The precipitate was collected by centrifugation (8000 rpm, 10 minutes) and washed with ethanol or deionized water. The precipitate was subsequently dispersed in ethanol containing 1% HCl and reacted at 60 °C for 6 h under stirring. Finally, the product was collected by centrifugation, washed with ethanol several times and dried in a vacuum oven at 60 °C overnight to obtain MSNs.

For the synthesis of MSNs loaded with Pt and Pd (MPP) and MPPs grafted with cinnamaldehyde (MPPC), 133 mg of MSNs was dissolved in 180 mL of methanol, which is presumed to act as a catalyst or support. A total of 20 mL of deionized water containing 0.1 mmol of chloroplatinic acid and 0.1 mmol of palladium chloride was introduced into the solution. The mixture was stirred via a magnetic stirrer at 70 °C and 400 rpm for 3 hours. After the reaction, the solvent volume was reduced via rotary evaporation until approximately 10 mL of methanol remained. The reaction mixture was transferred to a centrifuge tube and washed three times with methanol. Following the washing step, the solution was lyophilized via a freeze dryer to obtain the MPP nanozymes. Ultrasonically disperse the obtained MPP nanozymes in 20 mL of methanol via a water bath to ensure uniform dispersion. Two hundred microliters of 3-aminopropyltriethoxysilane were introduced into the dispersion, the container was sealed, and the mixture was stirred overnight in a metal bath at 65 °C. After the reaction, the solution was transferred into a 50 mL centrifuge tube and washed twice with methanol to remove any unreacted 3-aminopropyltriethoxysilane. After washing, the MPP nanozymes were further dispersed in methanol. Then, cinnamaldehyde was added in an amount equivalent to 1.1 times the molar amount of the amino silane coupling agent, followed by stirring. Upon completion of the reaction, the reaction mixture was transferred into a centrifuge tube and washed three times with methanol. Following the washing step, the solution was lyophilized to obtain MPPC nanozymes.

For MPP@CM and MPPC@CM, the 4T1 cell membrane was first obtained via the differential centrifugation method. First, the cells were collected by scraping. After being washed with PBS, the cells were resuspended in low-osmotic lysis buffer containing membrane protein extraction reagents and PMSF. After incubation in an ice bath, mechanical destruction was performed, followed by centrifugation at 1000 × g. Then, the supernatant was centrifuged at 15000 × g for 35 min to obtain the cell membrane. The cell membrane fragment suspension and MPP were mixed and allowed to coextrude through a 200 nm polycarbonate membrane to obtain MPP@CM. MPPC@CM was prepared by replacing MPP with MPPC.

**Characterization**

The morphology and elemental distribution were analyzed via transmission electron microscopy (TEM, FEI Tecnai F20). The surface chemical state was measured via X-ray photoelectron spectroscopy (XPS, Thermo Scientific K-Alpha). A Malvern Zetasizer Nano ZS90 was used for zeta potential and dynamic light scattering (DLS) analyses. Fourier transform infrared spectroscopy (FTIR, Thermo Fisher Scientific Nicolet iS20) was applied for measurement of the infrared spectral properties. The absorption spectrum was obtained via ultraviolet‒visible spectroscopy (UV‒vis, Shimadzu UV-3600i Plus).

**Enzyme-like activity measurements**

For the CAT-like activity measurements, H_2_O_2_ was mixed with MPP@CM or MPPC@CM NPs, and the concentration of O_2_ bubbles was measured via a dissolved oxygen meter to detect the generation of O2.

For OXD-like activity measurements, TMB was added to the MPP@CM or MPPC@CM solution. The oxidation of TMB was subsequently measured by recording the absorption variations at 652 nm.

For POD-like activity measurements, H_2_O_2_ solution (10 mM) containing TMB was added to MPP@CM or MPPC@CM. UV‒vis absorption spectra of the solutions at 652 nm were measured. The Michaelis‒Menten kinetic curves of MPP@CM and MPPC@CM were obtained by plotting the respective initial velocities against a series of MPP@CM and MPPC@CM concentrations.

For GSH consumption evaluation, DTNB was used as a probe, which can react with the sulfhydryl groups of GSH to form yellow 2-nitro-5-thiobenzoic acid (TNB), with a characteristic absorption peak at 412 nm. MPP@CM or MPPC@CM was mixed with GSH in PBS, and the supernatant was collected at various time intervals to detect sulfhydryl groups in GSH via DTNB. The resulting change in the absorbance of the solution at 412 nm was recorded via a UV–vis spectrophotometer.

ABTS was used to investigate the clearance of ROS from MPP@CM or MPPC@CM under the same conditions.

·OH and ·O_2_^-^ formation was confirmed via ESR spectroscopy. The ·OH/·O_2_^-^ radical generated by MPP@CM or MPPC@CM in the presence of H_2_O_2_ was measured via an E500 spectrometer (BRUKER) with DMPO as a spin-trapping agent.

**Accelerator**

An RS2000 160-kVp X-ray Biological Irradiator (Rad Source) was utilized for photon beam delivery.

**Cell culture**

The 4T1 murine breast cancer cell line and IEC-6 cell line were obtained from Immocell (Xiamen, China). The cells were grown in the recommended cell culture medium under normal (21% O_2_ and 5% CO_2_) or hypoxic (1% O_2_ and 5% CO_2_) conditions. The cells were cryopreserved via cell freezing medium (Life-iLab, China) and stored at -80 °C.

**Cell viability assessments**

A Cell Counting Kit-8 (CCK8) assay was used to measure cell viability. First, the cells were seeded in 96-well plates at a density of 5 × 10^3^ cells/well and incubated overnight. The following day, the cells were treated with various concentrations of MPP or MPPC. At the experimental endpoint, the cells were washed with warm PBS and incubated with cell culture medium containing CCK8 (Life-iLab, China). The optical density (OD) was measured at 450 nm to confirm the cell viability.

**Cell apoptosis analysis**

To detect cell apoptosis, 4T1 cells were inoculated into a 6-well plate at a density of 10^6^ cells/well and incubated overnight. Before irradiation, fresh medium containing MPP@CM or MPPC@CM (50 μg/mL) was added to the cells. After an additional 24 h, the cells were collected and dyed with Annexin V-FITC/PI (Abkine, China). Flow cytometry analysis was performed on a Beckman Cytoflex S.

**Western blot**

Protein lysates were separated by SDS‐PAGE and transferred to PVDF membranes (Millipore, USA). The membranes were subsequently blocked with 5% BSA in TBST for 1 h at room temperature, followed by incubation with anti-GPX4 antibody (Abcam, ab125066), anti-HIF-1α antibody (CST, #36169) and anti-β-actin antibody (CST, # 4967) at 4 °C overnight and incubation with HRP-conjugated secondary antibodies for 1 h at room temperature. The target protein signals were detected via enhanced chemiluminescence (ECL) reagents (Abkine, China).

**Confocal fluorescence imaging**

The generation of intracellular reactive oxygen species (ROS) was measured with a DCFH-DA probe (Beyotime, China, S0033M), and lipid peroxidation (LPO) was measured with a C11-BODIPY probe (Beyotime, China, S0043M). 4T1 cells were seeded in confocal dishes and incubated overnight. Then, the 4T1 cells were incubated with PBS, MPP@CM with RT (4 Gy), or MPPC@CM with or without RT (4 Gy) for 4 h. Next, the cell culture medium was replaced with fresh medium containing DCFH-DA or C11-BODIPY for 20 min, and the cells were washed twice with PBS. Confocal fluorescence imaging (CLSM) was subsequently used to observe ROS generation or LPO accumulation. Similar to the above steps, live/dead staining was performed with a Calcein/PI Cell Viability Kit (Beyotime, China, C2015M) followed by CLSM.

**Clonogenic assay**

4T1 cells were seeded in 6-well plates at densities of 500, 1000, 2000 and 4000 cells per well at doses of 0, 2, 4 and 6 Gy, respectively. Before irradiation, MPP@CM or MPPC@CM was added to the cell culture. After treatment, the cell culture medium was changed, and the cells were cultured for 2 weeks. The cell colonies were then imaged, and the number of colonies with >50 cells was counted.

**Intracellular MDA evaluation**

4T1 cells were lysed after treatment with PBS, RT, MPPC@CM, MPP@CM+RT or MPPC@CM+RT. After centrifugation, the supernatants were mixed with the working solution in an MDA kit (Solarbio, China, BC0025) and heated to 100 °C. The absorption of the supernatant at 532 nm was subsequently determined and recorded. Protein quantification with a BCA kit was used to normalize the MDA content.

**Consumption of GSH In Vitro**

To detect the in vitro GSH level, 4T1 cells were seeded in 6-well plates and incubated for 24 h. After treatment with MPPC@CM at various concentrations for 12 h, the cells were collected, and the intracellular GSH content was detected according to the instructions of the GSH detection kit (Solarbio, China, BC1175).

**γ-H_2_AX and CRT expression and HMGB1 extracellular release assays**

4T1 cells were seeded on a 24-well glass bottom cell culture dish at a density of 10^5^ per well for 24 h of incubation. After various treatments, the cells were washed with PBS twice, fixed with 4% paraformaldehyde, incubated with 5% BSA and then with an anti-γ-H2AX antibody (CST, #9718), anti-CRT antibody (CST, #12238) or anti-HMGB1 (CST, #3935) antibody overnight at 4 °C and an APC-conjugated secondary antibody for 2 h. The cell nuclei were stained with DAPI at room temperature for 10 min. The fluorescence signals of CRT and HMGB1 were observed via CLSM.

**Ex vivo DC maturation analysis**

Bone marrow-derived dendritic cells (BMDCs) were harvested from the bone marrow of the mice. 4T1 cells were prepared one day before treatment. The supernatant of the 4T1 cell medium containing different formulations was then added to the BMDCs. The nonadherent DCs were ultimately stained with the corresponding antibodies, and the proportion of mature DCs was analyzed via flow cytometry to assess DC maturation.

**Fluorescence imaging**

4T1 tumor-bearing mice were subjected to fluorescence imaging. When the tumor volume was approximately 300 mm^3^, the tumor-bearing mice were intravenously injected with MPPC or MPPC@CM. Near-infrared fluorescence images were obtained with an in vivo imaging system (IVIS) at 24 h postinjection.

**Biodistribution of MPP@CM and MPPC@CM in vivo**

Tumor-bearing mice received intravenous injections of MPPC or MPPC@CM. The main organs and tumors were collected at 24 h postinjection for biodistribution analysis via inductively coupled plasma atomic emission spectroscopy (ICP‒AES).

**Biocompatibility experiment**

Healthy BALB/c female mice (Hubei Biont Biological Technology) aged 6 weeks received intravenous injections of PBS, MPP@CM or MPPC@CM. The main organs and blood were collected and analyzed through H&E and blood tests at 14 d postinjection.

**Antitumor efficacy of MPPC@CM-mediated radiosensitization**

A total of 10^6^ 4T1 cells were subcutaneously injected into the right laps of female BALB/c mice aged 6 weeks. After the tumor volume reached approximately 200 mm^3^, the mice were divided into 5 groups and subjected to various treatments: (1) control, (2) RT, (3) MPPC@CM, (4) MPP@CM+RT, and (5) MPPC@CM+RT. The dosage of RT was 4 Gy. The mice in groups 3--5 received MPPC@CM (5 mg/kg) or MPP@CM (equivalent dose of 10 mg/kg MPP@CM). Tumor dimensions (length and width) and body weights were monitored and recorded every 2 days. At 14 days posttreatment, the mice were sacrificed, and the tumors were collected prior to fixation for pathological examination (GPX4, Ki67 immunofluorescence staining and TUNEL staining). Ki67 was stained with anti-Ki67 antibody (Abcam, ab 15580). All animal procedures were conducted in accordance with the procedures reviewed and approved by the Care of Experimental Animals Committee. All efforts were made to minimize animal suffering.

**Radiation Sensitization Synergy of PD-L1 Blockade**

A bilateral tumor model was established with 10^6^ 4T1 cells injected on the right lap and 10^5^ 4T1 cells injected on the left lap 7 days later. The mice were subsequently divided into four groups: (1) the control group, (2) the RT+aPD-L1 group, (3) the MPPC@CM+aPD-L1 group, and (4) the MPPC@CM+ RT+aPD-L1 group. The mice in groups 3--4 received MPPC@CM (10 mg/kg). The mice received an intraperitoneal injection of 75 μg/kg on days 3, 4 and 7. At the end of the observation period, the tumors and lymph nodes were collected and subsequently mechanically filtered into single cells, which were stained with CD11c, CD80, CD86, CD4, and CD8 antibodies (BioLegend, USA) for cytometry analysis. Serum was collected for ELISA analysis of IFN-γ, TNF-α, and IL-6. The tumor tissues were collected prior to fixation for pathological examination (CD8, CRT and HMGB1 immunofluorescence staining and HE staining).

**HE Staining**

Tissues from the mice were fixed in 10% formalin for 24 h, followed by processing and paraffin embedding. The paraffin sections were deparaffinized and stained with hematoxylin and eosin (HE). The sections were subsequently sealed with a neutral resin. The morphological changes were examined through the observation of pathological images under an inverted microscope.

**Statistics**

All the experimental data were repeated three or more times, and each experiment was independent of each other. The data were processed with GraphPad Prism 9 software, and the experimental results are presented as the means ± standard deviations (means ± SD). Student’s t test was used for comparisons between two groups, and one-way ANOVA was used for comparisons between multiple groups. *p < 0.05, **p < 0.01, ***p < 0.001. All statistical analyses were performed using GraphPad Prism (8.0).

**Supplementary Tables**

**Table S1.** CAT-like activity of MPP@CM and MPPC@CM.

|  | *K_cat_ (S^-1^)* | Specific activity (U) |
| --- | --- | --- |
| MPP@CM | 0.010438 | 125.26 |
| MPPC@CM | 0.004173 | 48.10 |

**Table S2.** OXD-like activity of MPP@CM and MPPC@CM.

|  | *K_cat_ (S^-1^)* | Specific activity (U) |
| --- | --- | --- |
| MPP@CM | 0.0574 | 0.0190 |
| MPPC@CM | 0.05528 | 0.0063 |

**Supplementary Figures**


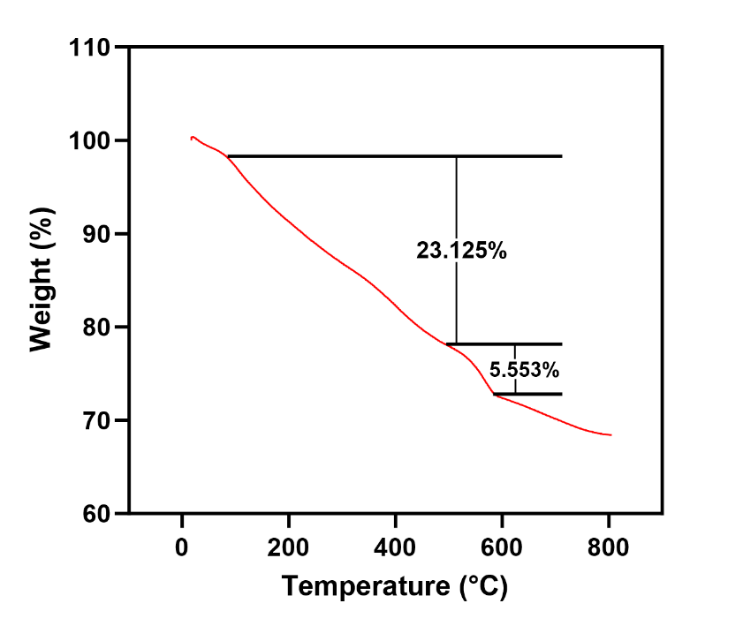


**Figure S1.** TGA curve of MPPC.

**
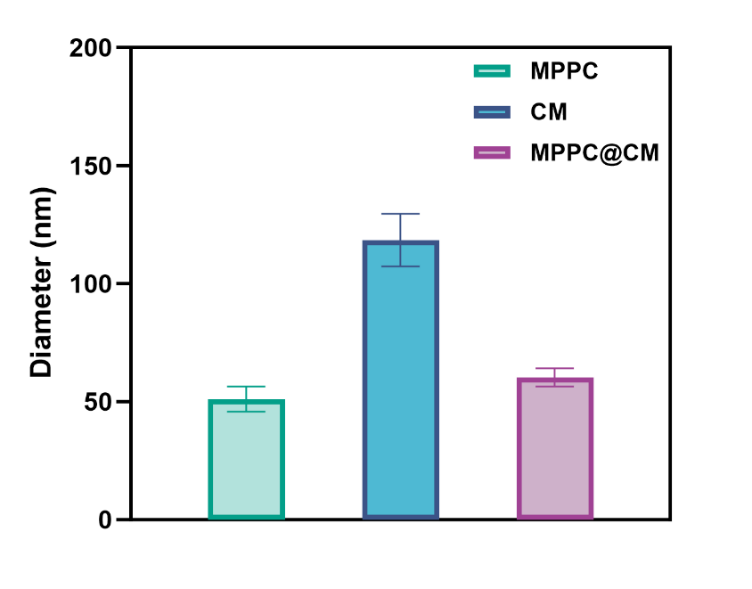
**

**Figure S2.** Diameter distribution of MPPC, CM and MPPC@CM. Results were presented as the mean ± SD from three independent experiment.

**
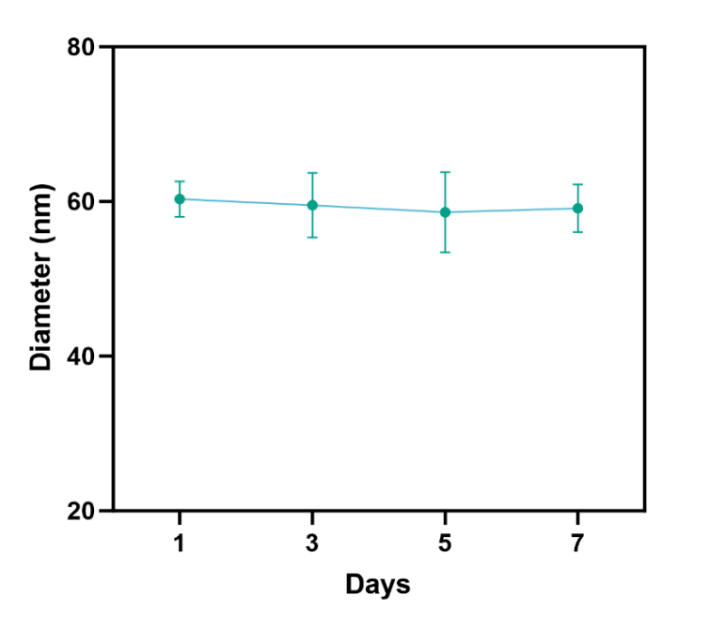
**

**Figure S3.** Stability evaluation of MPPC@CM in PBS over 7 days. Results were presented as the mean ± SD from three independent experiment.


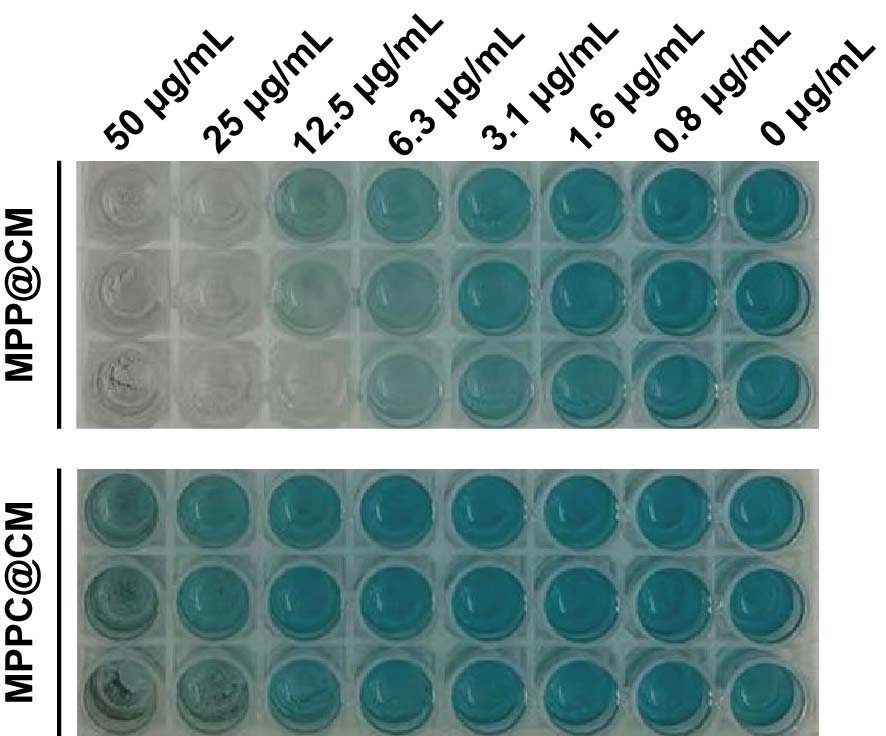


**Figure S4.** Colorimetric assays of the POD-like activity of MPP@CM and MPPC@CM at various concentrations using ABTS.


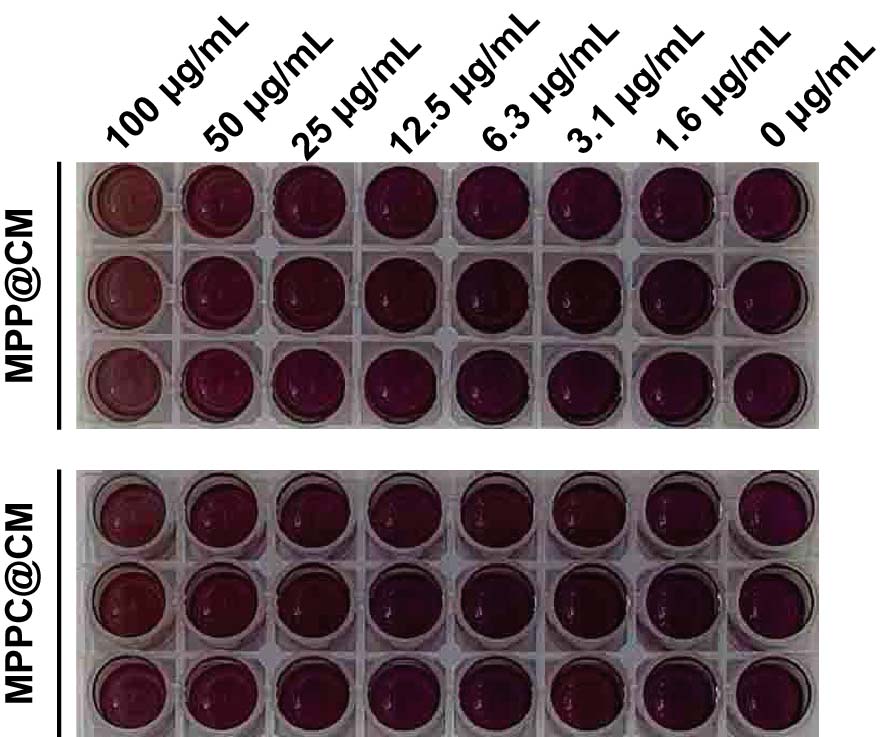


**Figure S5.** Colorimetric experiments of the POD-like activity of various concentrations of MPP@CM and MPPC@CM were performed via DPPH.


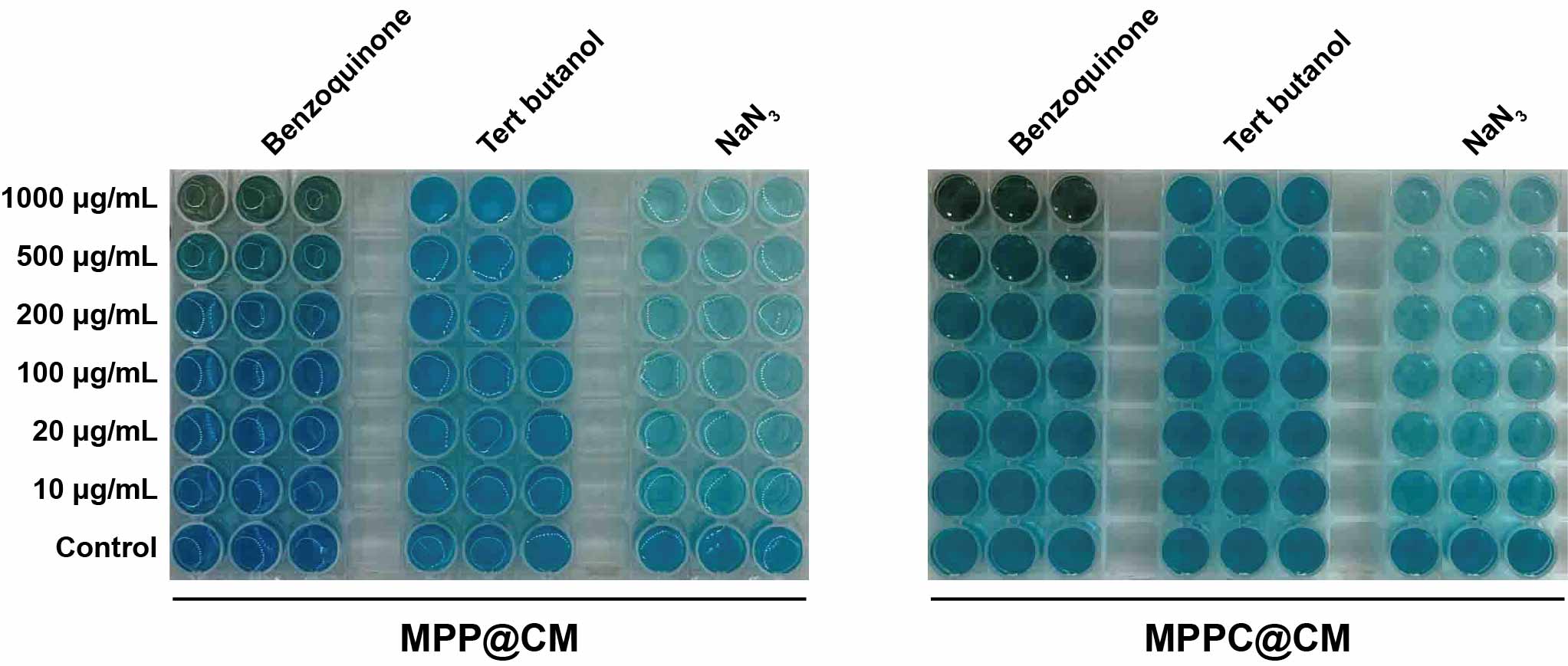


**Figure S6.** Colorimetric experiment of the POD-like activity of MPP@CM and MPPC@CM using the singlet oxygen scavenger benzoquinone, the hydroxyl radical scavenger tert-butanol and the quencher of the superoxide radical NaN_3_.


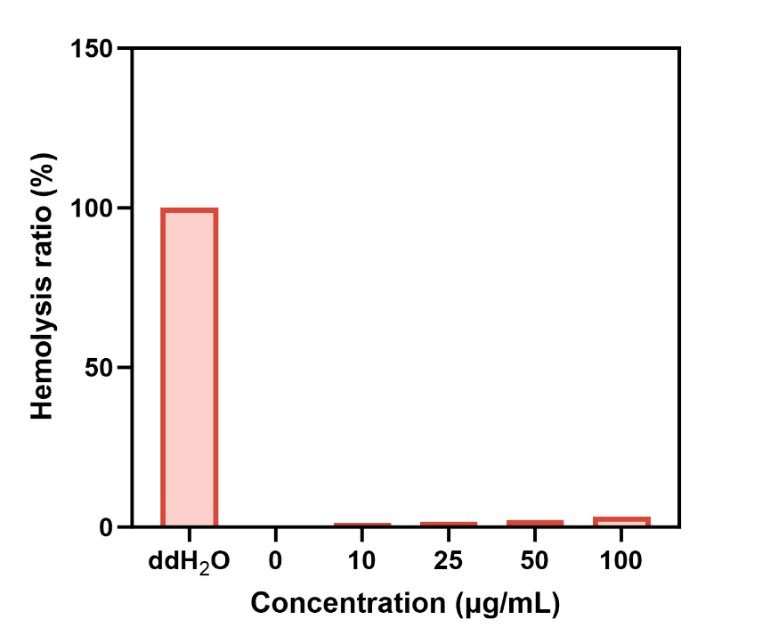


Figure S7. Hemolysis assay of MPPC@CM.


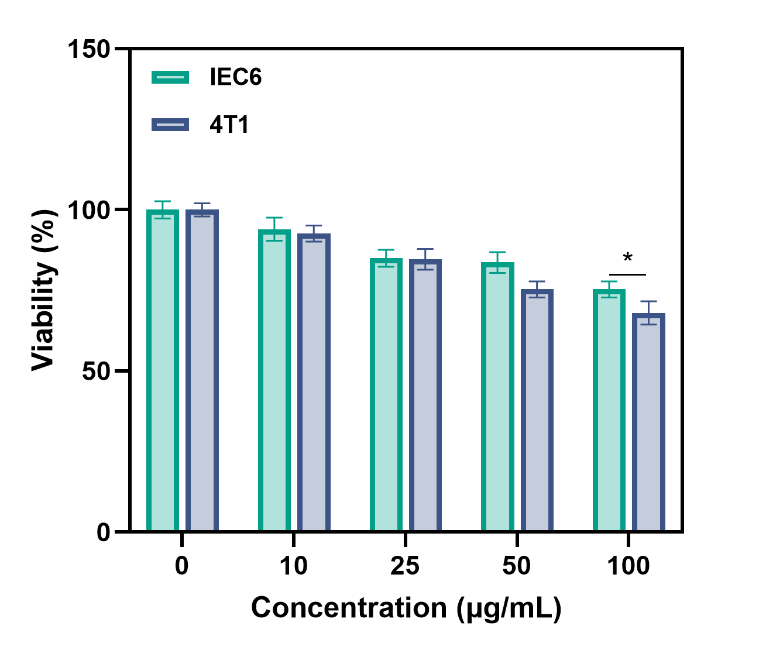


**Figure S8.** Viability of 4T1 and IEC6 cells coincubated with MPP@CM. Two-way ANOVA with Tukey’s post hoc test, *p < 0.05. Results were presented as the mean ± SD from three independent experiment.


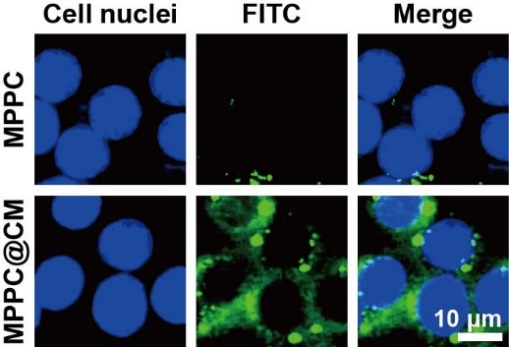


**Figure S9.** CLSM image of cellular uptake (Scale bar: 10 μm).


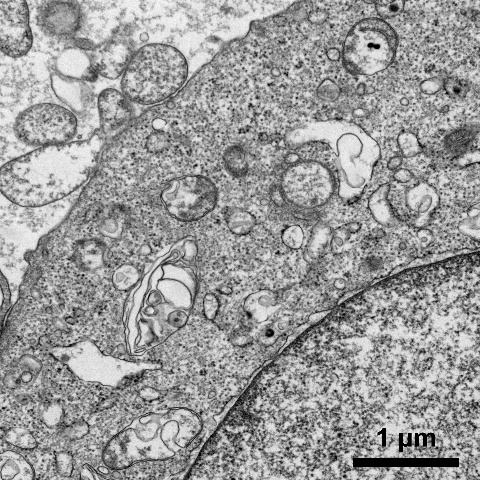


**Figure S10.** TEM images of 4T1 cells in MPPC@CM+RT group.


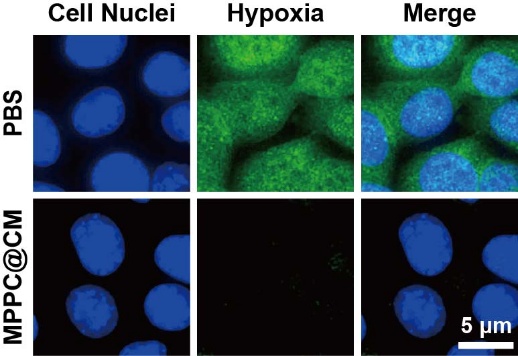


**Figure S11.** CLSM images of HIF-1α in 4T1 cells treated with PBS or MPPC@CM.


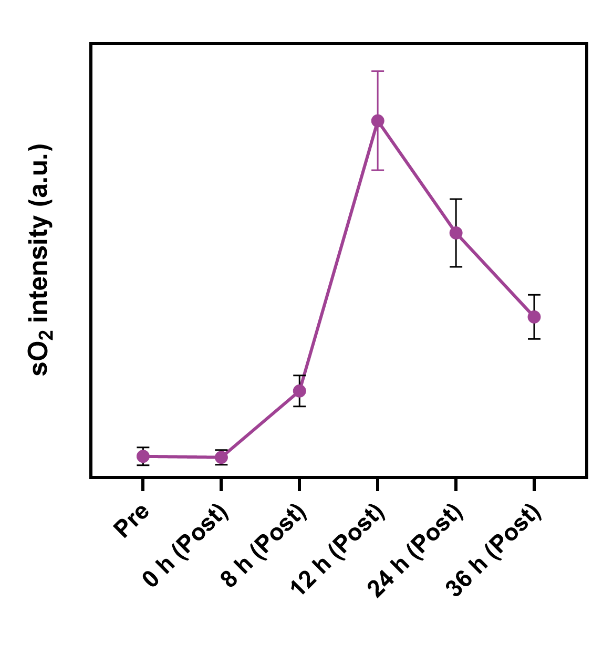


**Figure S12.** Quantitative analysis of intratumoral sO_2_ levels at various time points. Results were shown as mean ± SD from 5 mice of each group.


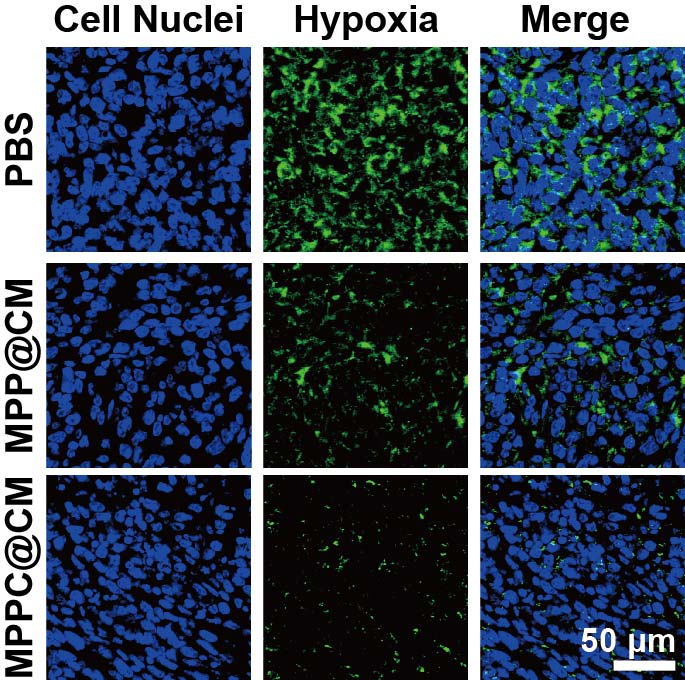


**Figure S13.** CLSM images of HIF-1α immunofluorescence staining (scale bar: 50 μm).


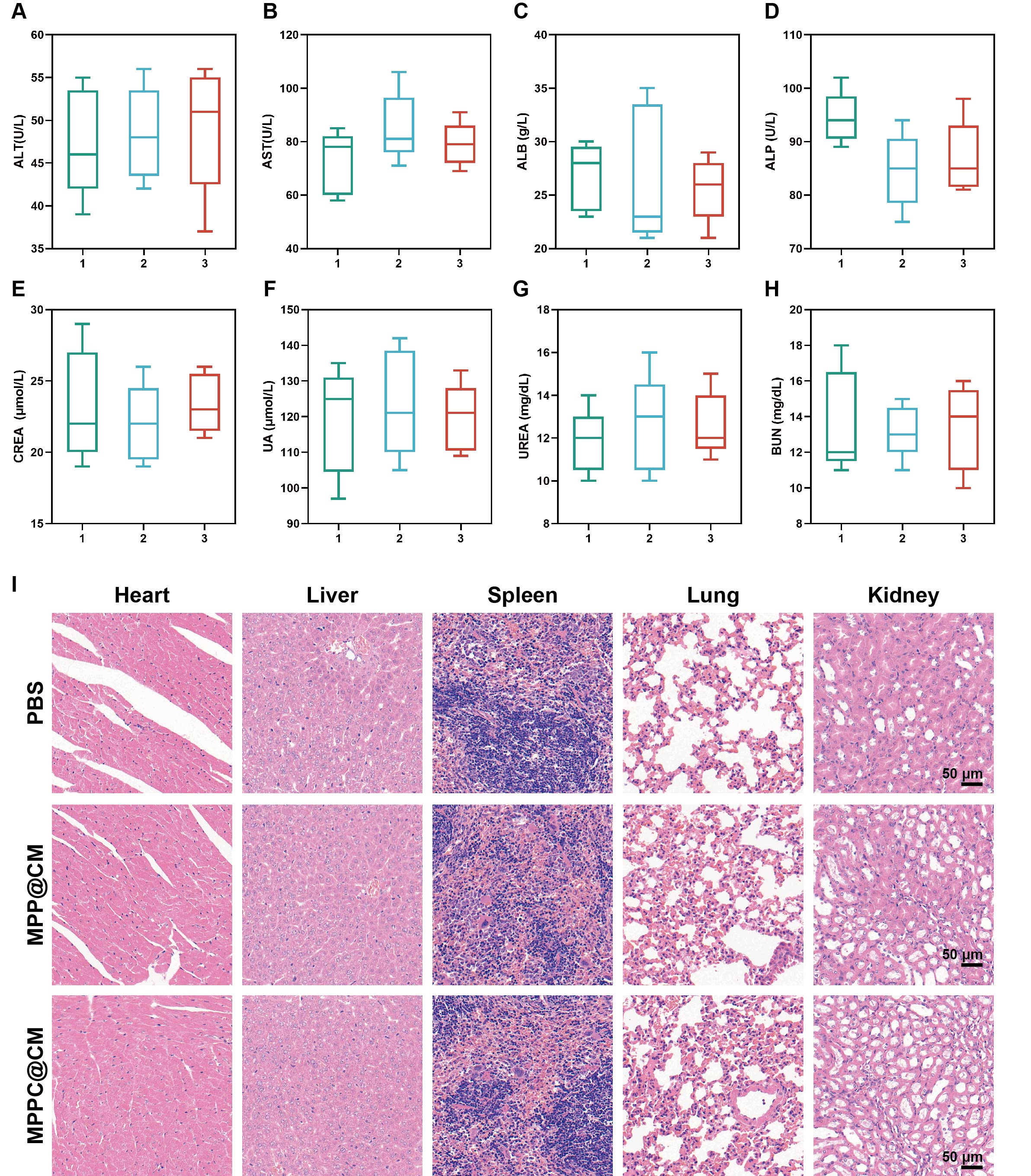


**Figure S14.** Biosafety evaluation. (A-H) Routine blood and biochemical analyses of the liver and kidney. Results were shown as mean ± SD from 5 mice of each group. (I) HE staining of main organs from mice that received various treatments (scale bar: 50 μm).


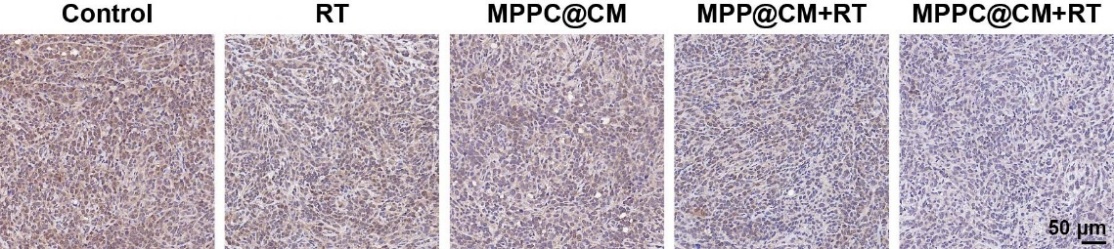


**F****igure S15.** Immunohistochemical staining of GPX4 in tumors from various treatment groups.


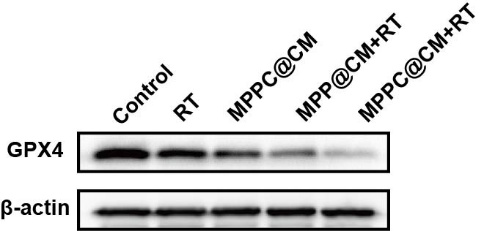


**Figure S16.** Western blot analysis of tumors across different treatment groups.

**
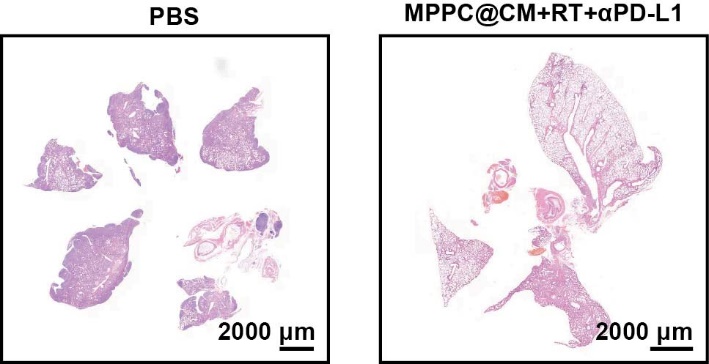
**

**Figure S17.** HE staining of lungs from different treatment groups (scale bar: 2000 μm).
